# Intron retention and rhythmic diel pattern regulation of carotenoid cleavage dioxygenase 2 during crocetin biosynthesis in saffron

**DOI:** 10.1007/s11103-016-0473-8

**Published:** 2016-04-12

**Authors:** Oussama Ahrazem, Angela Rubio-Moraga, Javier Argandoña-Picazo, Raquel Castillo, Lourdes Gómez-Gómez

**Affiliations:** Departamento de Ciencia y Tecnología Agroforestal y Genética, Facultad de Farmacia, Instituto Botánico, Universidad de Castilla-La Mancha, Campus Universitario s/n, 02071 Albacete, Spain; Fundación Parque Científico y Tecnológico de Castilla-La Mancha, Campus Universitario s/n, 02071 Albacete, Spain; VITAB Laboratorios, Polígono Industrial Garysol C/Pino, parcela 53, La Gineta, 02110 Albacete, Spain

**Keywords:** Apocarotenoid, CCDs, Crocetin, Intron retention, Promoter, Saffron, Splicing

## Abstract

The carotenoid cleavage dioxygenase 2, a new member of the CCD family, catalyzes the conversion of zeaxanthin into crocetin-dialdehyde in *Crocus*. *CCD2* is expressed in flowers, being responsible for the yellow, orange and red colorations displayed by tepals and stigma. Three *CsCCD2* genes were identified in *Crocus sativus*, the longest contains ten exons and the shorter is a truncated copy with no introns and which lacks one exon sequence. Analysis of RNA-seq datasets of three developmental stages of saffron stigma allowed the determination of alternative splicing in *CsCCD2*, being intron retention (IR) the prevalent form of alternative splicing in *CsCCD2*. Further, high IR was observed in tissues that do not accumulate crocetin. The analysis of one *CsCCD2* promoter showed *cis*-regulatory motifs involved in the response to light, temperature, and circadian regulation. The light and circadian regulation are common elements shared with the previously characterized *CsLycB2a* promoter, and these shared common *cis*-acting elements may represent binding sites for transcription factors responsible for co-regulation of these genes during the development of the stigma in saffron. A daily coordinated rhythmic regulation for *CsCCD2* and *CsLycB2a* was observed, with higher levels of mRNA occurring at low temperatures during darkness, confirming the results obtained in the in silico promoter analysis. In addition, to the light and temperature dependent regulation of *CsCCD2* expression, the apocarotenoid β-cyclocitral up-regulated *CsCCD2* expression and could acts as a mediator of chromoplast-to-nucleus signalling, coordinating the expression of *CsCCD2* with the developmental state of the chromoplast in the developing stigma.

**Electronic supplementary material:**

The online version of this article (doi:10.1007/s11103-016-0473-8) contains supplementary material, which is available to authorized users.

## Introduction

Carotenoids are widespread highly hydrophobic compounds that have been isolated from many living organisms. However, their biosynthesis is restricted to plants, some bacteria and fungi. In plants, yellow, orange, and red colours provided by carotenoids accumulate in the chromoplasts of flowers and fruits. In these tissues, carotenoids act to attract pollinators and agents of seed dispersal. In addition to carotenoids, their oxidative and enzymatic cleavage products called apocarotenoids, are crucial for various biological processes in plants, such as stress responses, regulation of growth and development, and in the interaction with their environment (Al-Babili and Bouwmeester 2015; Walter and Strack 2011). For humans, some apocarotenoids synthesized by plants have an important economic value, due to their very potent aroma (e.g. β-ionone and safranal) and their pigmentation capacity (e.g. crocin and bixin) (Gómez-Gómez et al. 2010; Winterhalter and Rouseff 2001).

Whereas carotenoid biosynthesis is limited to plants, some bacteria and fungi, apocarotenoids are present in practically all organisms. A group of enzymes called carotenoid cleavage dioxygenases are responsible for catalyzing the cleavage reactions on carotenoids (Sui et al. 2013). In plants, there are two different groups of carotenoid cleavage dioxygenases (CCDs), the NCED enzymes catalyze the first step towards abscisic acid (ABA) biosynthesis (Schwartz et al. 1997) and the CCD enzymes that have more divergent activities and catalyze the cleavage of a variety of carotenoid substrates at specific double bond positions (Auldridge et al. 2006). NCEDs and CCDs enzymes belong to an ancient family that employs a nonheme iron cofactor to activate molecular oxygen for insertion into a carbon–carbon double bond of the carotenoid polyene. Five subfamilies have been defined in the CCD plant family; CCD1, CCD2, CCD4, CCD7 and CCD8, which differ in their substrate and regioselectivity. Members from the CCD1, CCD4, CCD7 and CCD8 subfamilies have been identified in all the high plant genomes sequenced up to date (Ahrazem et al. 2010b), being CCD2 an exception, with members only identified in *Crocus sativus* and in other *Crocus* species (Ahrazem et al. 2016; Frusciante et al. 2014). The CCD2 enzymes are closely related to the CCD1 subfamily. But while CCD1 enzymes showed a broad substrate and double bond specificity (Ilg et al. 2014; Walter and Strack 2011), CCD2 enzymes displayed a restricted specificity on substrates and double bond recognition (Frusciante et al. 2014). CCD2 has been identified in *C. sativus* (CsCCD2) as the enzyme responsible for the cleavage of zeaxanthin at 7,8(7′,8′) positions to produce crocetin dialdehyde (C20) and hydroxy-β-cyclocitral (C10). The enzyme also cleaves lutein as well as 3-OH-β-apocarotenals at the 7,8 position, suggesting the requirement for a 3-OH-β-ring at the proximal end of the substrate molecule (Frusciante et al. 2014). In addition, CCD1 and CCD2 enzymes also differ in their location, while CCD1 enzymes are cytosolic, CCD2 enzymes are localized in plastids (Ahrazem et al. 2016).

The flower of *C. sativus* has a long red divided stigma, characterized for a high content in apocarotenoids that when processed constitute the saffron spice, one of the oldest spices used as flavouring and colouring agent (Ahrazem et al. 2015b). These apocarotenoids are the products of CCD2 activity, crocetindialdehyde and hydroxy-β-cyclocitral, which are the substrates of aldehyde dehydrogenases and the resulting products are further glucosylated generating crocins and picrocrocin, which after thermal degradation is transformed into the odour-active volatile safranal (Moraga et al. 2004, 2009). Crocins and safranal are responsible, respectively, of the colour and aroma properties of the saffron spice.

The accumulation of carotenoid precursors and saffron’s apocarotenoids in *C. sativus* stigmas have been previously studied (Castillo et al. 2005; Moraga et al. 2009; Rubio et al. 2008), and it has been shown that transcriptional regulation of two chromoplast-specific carotenogenic genes, β-carotene hydroxylase (*CsBCH1*) and the stigma-specific lycopene cyclase (*CsLycB2a*), are controlling the carotenoid pool for apocarotenoid biosynthesis (Ahrazem et al. 2010a). As carotenoids, crocins and picrocrocin accumulation in saffron stigma are spatially and temporally regulated. These apocarotenoids begin to accumulate in the stigma at very early developmental stages (Moraga et al. 2009), coincident with the expression levels of *CsCCD2* (Frusciante et al. 2014; Rubio et al. 2008), which levels decline when the stigma is fully developed and the apocarotenoid levels have reached a plateau, starting thereafter its mobilization from the senescent stigma (Rubio-Moraga et al. 2010). The regulatory mechanisms that control carotenoid and apocarotenoid accumulation remain poorly understood in saffron. In fact, to date no regulatory genes directly controlling carotenoid or apocarotenoid biosynthetic gene expression have been isolated. Nonetheless, it is known that a major driving force for apocarotenoids production in saffron is the transcriptional regulation of genes encoding lycopene cyclase, *CsLycB2a*, the enzyme catalyzing zeaxanthin production, *CsBCH1*, and *CsCCD2*. Consistently, the burst in apocarotenoids biosynthesis that occurs in the stigma of saffron is correlated with a very fast up-regulation of *CsLycB2a* and *CsCCD2* transcripts (Ahrazem et al. 2010a; Frusciante et al. 2014). Thus, the developmental and concerted regulation of carotenogenesis and crocetin biosynthesis in saffron stigma suggest the presence of commom *cis*-regulatory elements in the promoter regions of these genes.

In this article, we address the evolutionary relationships of CsCCD2 with others plants CCDs by examination of gene architecture. For such a purpose, the genomic sequences and organization of *CsCCD2* are described, and these sequences were compared to *CCD1* sequences from angiosperms and gymnosperms in the databases. Taking advantage of transcriptome data from early developmental stigma stages and other saffron tissues, we identify differences in *CsCCD2* transcripts. We found that tissues that do not accumulate crocetin exhibited increased levels of intron retention in *CsCCD2* transcripts relative to stigma tissue. Further, a *CsCCD2* promoter region was isolated and compared with the previously identified *CsLycB2a* promoter and a new mechanism controlling the expression during stigma development and crocetin accumulation is discussed.

## Materials and methods

### Plant material and treatments

Corms of *C. sativus* donated by the Fundación Valeriano González (Albacete, Spain,) were used throughout the experiments. *C. ancyrensis* and *C. cartwrightianus* were collected from plants growing in the Botanical Garden of CLM (Albacete, Spain). Stigmas were collected at the developmental stages previously described (Rubio et al. 2008) and frozen in liquid nitrogen and stored at −80 °C until required.

To determine the regulation of *CsCCD2* three sets of experiments were designed. The first experiment was conducted to investigate the day/night regulation of *CsCCD2*. Stigmas in the orange stage (Rubio et al. 2008) were collected at different times from 16th to 18th October 2015, from 100 plants growing under field conditions. Humidity and temperature in the growing area were recorded during the selected period. In the second set of experiments, 100 saffron corms were placed on individual pots; 50 pots were covered simulating complete dark conditions while the other 50 pots were uncovered. Stigmas at different developmental stages from yellow, orange and red were collected at the same time from both batches. The third set of experiments was designed to determine the temperature and light regulation of *CsCCD2*. Flowers buds with yellow, orange and red stigmas were collected from four independent biological replicates and were placed in 24-well plates containing 1 ml distillate water. One plate was placed during 24 h at 37 °C, other at 8 °C, other at 20 °C in continuous light, other at 20 °C in continuous dark and the last one in a chamber at 20 °C with 12 h light/dark cycles, this last treatment was used as control. Further an additional batch of stigmas growing in control conditions were treated with β-cyclocitral (0.1 µl of a pure compound) during 24 h.

### Isolation of promoter sequence

Genomic DNA was prepared from *C. sativus* leaves by using the i-genomic Plant DNA extraction kit (iNtRON Biotechnology, Sangdaewon-Dong, Korea). The *CsCCD2* upstream flanking sequences were isolated with the GenomeWalker Universal Kit (BD Biosciences, Palo Alto, CA, USA). The genomic DNA was digested with four alternative restriction enzymes (*Dra*I, *Eco*RV, *Stu*I and *Pvu*II) and separately ligated with the adaptors provided by the manufacturer to obtain four restriction fragment libraries. PCRs were carried out using gene-specific primers (Table 1) based on the cDNA sequence obtained and adapter primers (AP1 and AP2). All PCR reactions were performed using Advantage 2 Polymerase mix (BD Biosciences, Palo Alto, CA, USA). The reaction products were ligated to pGEM-T with the TA Cloning Kit (Promega Corporation, Madison, USA). The ligated DNA was transformed into *E. coli* strain JM109. For each amplification round, 20 colonies were picked individually, amplified and the plasmid DNA extracted using a DNA Plasmid Miniprep Kit (Promega, Madison, WI, USA). Plasmids were sequenced using an automated DNA sequencer (ABI PRISM 3730xl, Perkin Elmer) from Macrogen Inc. (Seoul, Korea). Promoters were analyzed by the PLACE (Higo et al. 1999) and PlantCARE (Lescot et al. 2002) databases.

**Table 1:** Primer sequences used for *CsCCD2* analysis

Used for		5′ → 3′
RACE-5′ and 3′ reactions	F-1	TGAGTTGGGACCTAGAAGATATG
	F-2	TCTGAGGTCAATGTCATCGATG
	R-1	CCACTTCCACTGGTTGGAAATAC
	R-2	CAGGTCCATGAAGATGGCATAG
cDNA amplification	ATG1-F	CTTGACATGGAATCTCCTACTAC
	ATG2-F	GTATCAATGGCAAATAAGGAG
	R	TGTCTCTGCTTGGTGCTTCTGA
Promoter isolation and analysis	Rp1	GTAGCGGCCCAAATTTACGTGGA
	Rp2	TGGAAGGAAGATGGAGGAGCTCT
	Rp3	TGTGGTGTACAAACACCTGCATCC
	Rp4	TTACACGTTAGGCAAGATCGTCCA
	Rp5	CATTTCGGTGGCCCTGTTCGT
	Fp	AGGTGATCATAGGTACTCCCTA
gDNA amplification and analysis	F	TCTTGACATGGAATCTCCTACTACT
	F0	CAGTAGTATCAATGGCAAATAAGGAG
	F1	CGTCGCAGTGGACTTACTCGA
	F2	AACCCCAAGTTTGCTCCCGTC
	F2b	CGTCACGGTTTAAACAAGAAG
	F3	ATACTAGTACTTCGAACATTGA
	F4	TGCCAACCTGGCATCAAATCT
	F5	TGATGAGGATGACGGTTACTTGA
	F1-gw-3′	TCTGAGGTCAATGTCATCGATGCA
	F2-gw-3′	CAGAACCTGTGGCTGTTGTGGAAC
	FH	ATGCTTGAATGGAGTGTTTCTGA
	F(E + I4)	CGATCCGTTAACTGGTAATTCCT
	R(E + I1)	ATCTGCAGGTCGAGTATAGGAGA
	R1	TTGGGGTTAGGGCCAACTCTC
	R2	TAAGCAATCCTCTTAGATCTCCA
	R3	GGAGGCGAGAGGGAATATCCA
	R4	TGTCATGTCTCTGCTTGGTGCT
	R5	TGGAAGCAACCGATACGAGCT
	R6	TGCATCGATGACATTGACCTCA
	R7	TCTCCAATCTGCAGGTCGAGTA
	R8	CCATCTTTAATACGCAATCCATGA
	RH	ATCAGGACTCTCAAGGCGACA
For PET28 cloning and activity assays	F-Pet28	GACGGAGCTCGAATTCTCATGGAATCTCCTACTACT
	R-Pet28	TCGCGGATCCGAATTCTCATGTCTCTGCTTGGTGC
cDNA amplification *CancyCCD2*	R	CTACGTATTCTTAGAGACTAGT
	F	TCTGACAGACCATTGTAAACGGA
cDNA amplification *CcaCCD2*	R	AGTCGAAGCCCCGTTACTATCT
	F	GACATGGAATCTCCTACTACTAA

### Characterization of the genomic sequences

To determine the exon–intron boundaries of *CsCCD2*, the full-length genomic regions were isolated from *C. sativus* genomic DNA using PCR. The reaction was performed with Takara LA Taq polymerase (Takara Bio, Shiga, Japan) according to the manufacturer’s instructions using the primer sets detailed in Table 1. The PCR condition was: 94 °C for 1 min; 30 cycles of 98 °C for 15 s, 68 °C for 10 min; and 72 °C for 10 min. The amplified PCR fragments were subcloned into pGEM-T vector (Promega Corporation, Madison, WI, USA) and sequenced using an automated DNA sequencer (ABI PRISM 3730xl, Perkin Elmer) from Macrogen Inc. (Seoul, Korea).

### Phylogenetic analysis

The amino acid sequences of the selected proteins were aligned using the BLOSUM62 matrix with the ClustalW (http://www.clustal.org) algorithm-based AlignX module from MEGA Version 6.0 (http://www.megasoftware.net/mega.html). The alignments were executed by MEGA Version 6.0 to generate a Neighbour Joining Tree with bootstrapping (5000 replicates) analysis and handling gaps with pairwise deletion.

### Isolation of CsCCD2 cDNAs and analysis of protein sequences

Total RNA and mRNA were isolated from saffron stigma at different developmental stages by using AmbionPolyAtrack (Ambion, Inc.). First-strand cDNAs were synthesized using the SMARTer™ RACE cDNA Amplification Kit (Clontech, Palo Alto, CA) and oligo dTprimers (Promega, Madison, WI). These cDNAs were used as templates for RACE-5′ PCR and for *CsCCD2* cDNA cloning using specific oligonucleotides (Table 1). The amplified PCR products were cloned into pGEM-T (Promega Corporation, Madison, WI, USA) and transformed into *E. coli* strain JM109. The clones isolated were sequenced as described before.

All the CCD proteins were modelled using the Phyre2 server (http://www.sbg.bio.ic.ac.uk/phyre2/) (Kelley et al. 2015). For comparative modelling CsCCD2 proteins were aligned with the enzyme VP14, the crystal structure of which has been resolved (Messing et al. 2010).

### Activity assays in *E. coli*

The full-lengths of *CsCCD2*-*t* and *CsCCD2* cDNAs were cloned into the *Eco*RI site of the expression vector pET28 (Invitrogen, Carlsbad, California, USA) by means of In-Fusion cloning technology (Clonthec, Mountain View, California, USA) and using gene-specific primers (Table 1). The recombinant clones were transformed in *E. coli* Origami strain (Novagen, Darmstadt, Germany) engineered with a plasmid for the production of zeaxanthin (Cunningham and Gantt 1998). Transformed cells were cultured overnight at 37 °C in 3 ml LB medium supplemented with kanamycin (50 µg ml^−1^) and chloramphenicol (60 µg ml^−1^). The cultured cells were transferred to 50 ml LB medium supplemented with kanamycin (25 µg ml^−1^) and chloramphenicol (30 µg ml^−1^) and further cultured at 37 °C until an OD600 of 0.8 was reached. Cells were then induced with 0.5 mM IPTG and grew overnight at 20 °C. Cells harvest and pigment extractions and HPLC conditions were performed as previously described (Ahrazem et al. 2016).

### Expression analysis

Total RNA was extracted as described above, and gene-specific primers for real-time PCR are presented in Table 1. Transcript levels of *CsCCD2* were normalized with that of *RPS18* (Rubio-Moraga et al. 2014a), and each RNA sample was assayed in triplicate. The cycling parameters of qPCR consisted in an initial denaturation at 94 °C for 5 min; 40 cycles at 94 °C for 20 s 58 °C for 20 s and 72 °C for 20 s; and a final extension at 72 °C for 5 min. Assays were conducted in a StepOne™ Thermal Cycler (Applied Biosystems, Foster City, California, USA) and analyzed using StepOne software v2.0 (Applied Biosystems, Foster City, California, USA). DNA melt curves were created for each primer combination to confirm the presence of a single product.

### Transcript processing

We used transcript profiling analysis to investigate splicing as a key posttranscriptional RNA maturation step in *CsCCD2*. For that purpose we used RNA purified from stigmas at three different stages of development (white, yellow and orange). Total RNA was extracted with Trizol (Invitrogen Life Technologies Foster City, CA, USA) and treated with DNase RNase-Free Set (Qiagen, Valencia, CA, USA), according to the manufacturer’s instructions. We extracted RNA from a pool of ten independent biological replicates dissected from *C. sativus* buds. The concentration of RNA was measured in a NanoDrop ND-1000 spectrophotometer (NanoDrop Technologies, Wilmington, DE). A total of 5 µg of RNA from were sent to Macrogen Inc. (South Korea) for sequencing using the Genome AnalyzerIIx platform (Illumina Inc.) according to Illumina’s protocols. The cDNA libraries were dissipated onto an Illumina single-end flow cell formed by eight lanes using the Illumina Cluster Station (Illumina Inc.). One lane was used per sample of white, yellow and orange stages. The 101 bp reads obtained were collected using the Illumina GA II and sequencing-by-synthesis technology. The relative abundance of the transcripts was measured with the Cufflink software. This software measures the transcripts abundance as RPKM (Reads PerKilobase of exon model per Million mapped reads). These sequences were used to search for *CsCCD2* related sequences.

### Extraction and analysis of crocins by HPLC–DAD

Sample processing and analysis was done as previously described (Moraga et al. 2009) with modifications. Stigmas were ground in liquid nitrogen with a mixer mill MM400 (Retsch GmbH, Haan, Germany) in a 1.5 ml Eppendorf tube, and extracted with 1 ml Tris–HCl (50 mM, pH 7.5) (containing 1 M NaCl), and incubated on ice for 10 min followed by centrifugation at 3000*g* for 10 min at 4 °C. The supernatant was concentrated using a Speed Vac and the dried residues were stored at −80 °C until analysis by HPLC. All assays were performed in triplicate. The HPLC method used for the analysis and detection of crocins have been previously described (Castillo et al. 2005; Rubio Moraga et al. 2013).

## Results

### Exon–intron structure of CsCCD2

**Fig. 1:**
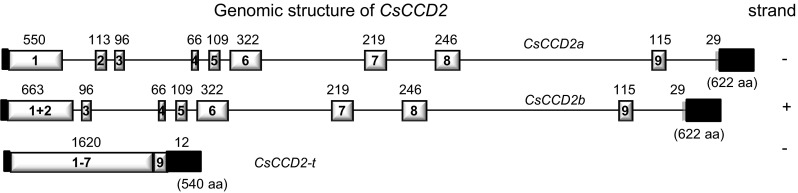
Analysis of the exon–intron structures of *CsCCD2* genes. Schematic diagram of the exon–intron structures of *CsCCD2* genes. *Gray* and *black boxes* indicate exons and untranslated regions (UTRs), respectively. Introns are indicated by *lines*. The size of each exon is indicated over the exon *boxes* in base pairs and the size of the protein products in presented on the *left* of each gene in parenthesis

**Fig. 2:**
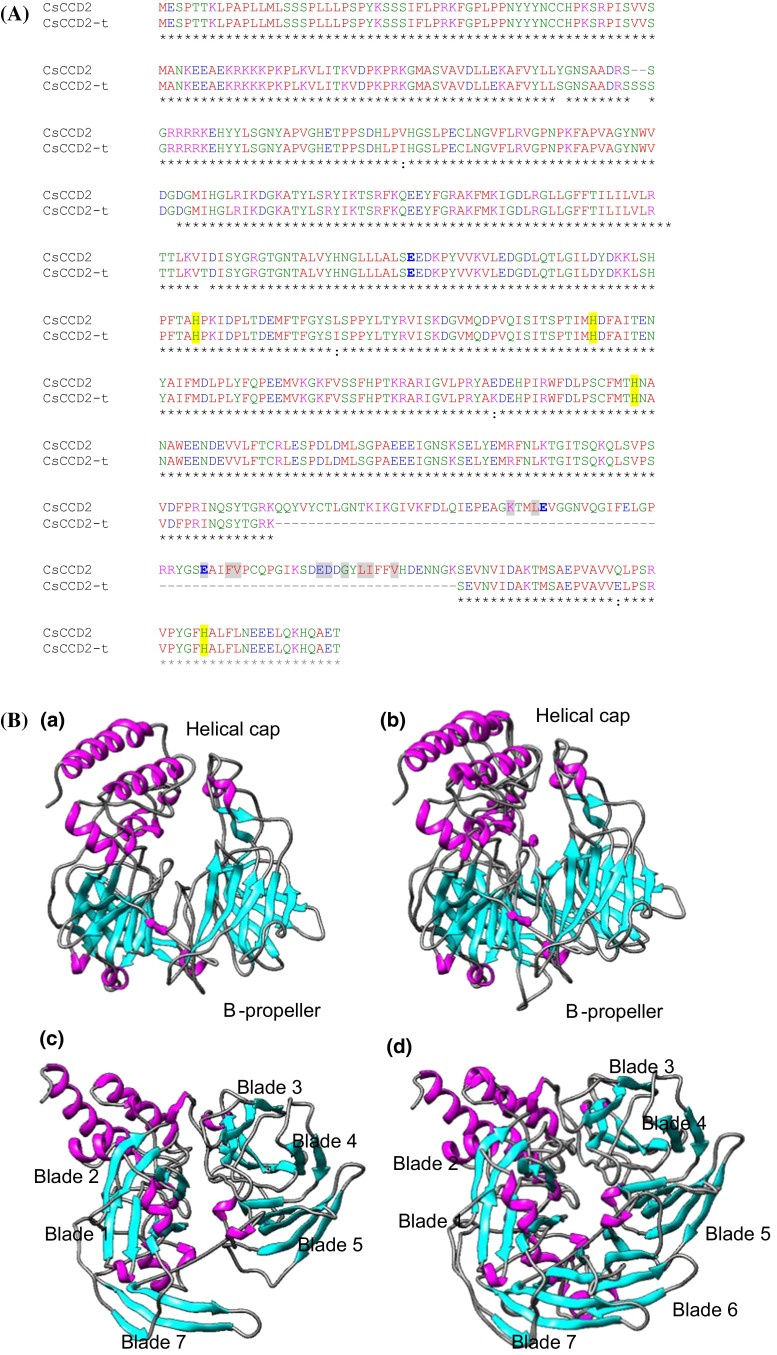
Amino acid sequences and structural comparisons of CsCCD2 and CsCCD2-t. **a** Amino acid alignment showing the conserved histidine residues highlighted with *yellow shading* and in *grey shading* the key amino acid residues necessary for CCDs catalytic activity. **b** Tridimensional models of CsCCD2-t (*a*, *c*) and CsCCD2 (*b*, *d*), with β-strands shown in cyan, α-helices in magenta, and loops in black

We determined the exon–intron boundaries, exon and intron sizes, and intron placement of *CsCCD2* genes on the genome of *C. sativus* by sequence alignment of *CsCCD2* cDNA with the isolated gDNA sequences. Three different *CsCCD2* genes were amplified from the genomic DNA of saffron and its structure determined by comparison with the *CsCCD2* cDNA clones (Supplemental Fig. S1, Fig. 1). The longest gene contained nine introns and ten exons and was named as *CsCCD2a*, followed in size by a gene with nine exons and eight introns, named as *CsCCD2b*. The third *CsCCD2* gene identified was identified as a truncated gene without introns, which lacks exon 8 and was named *CsCCD2*-*t* (Fig. 1). This gene encodes a protein of 480 amino acids that shows the absence of an important region for CCDs activity (Fig. 2a, b). Although the overall amino acid sequence identity among the CCDs enzymes of the different families is variable, there are consensus regions of absolute sequence conservation including the four fully conserved, iron-containing His residues. Three of these His residues hydrogen bond to a set of three conserved Glu residues (Sui et al. 2013) (Fig. 2a), which form the second coordination sphere with the ferrous ion. Although all the His residues are present in the CsCCD2-t protein, two Glu residues are missing in the protein (Fig. 2a). Mutagenesis studies on these residues indicate that are absolutely required for CCD catalytic function (Poliakov et al. 2005; Redmond et al. 2005; Takahashi et al. 2005). CCDs fold as a seven-blade β-propeller motif with several α-helical inserts that form an α-helical domain on top of the β-propeller (Kloer and Schulz 2006) that houses the active site. The iron ion cofactor is located close to the top face on the propeller axis and is coordinated by four conserved His residues and three second shell Glu residues. Each blade of the β-propeller contributes a single residue to the iron coordination system. This structure is maintained in CsCCD2, but in CsCCD2-t the β-propeller lacks blade 6 and creates a more open structure, resulting in an inappropriate “sealing” of the propeller structure (Fig. 2b). In order to determine whether CsCCD2-t was active or not, we tested its activity by expressing CsCCD2-t in *E. coli* strains engineered to accumulate zeaxanthin. When expression of CsCCD2-t was induced in the bacterial cells, accumulation of zeaxanthin was not affected and we were unable to identify any cleavage product by HPLC analysis compared with the activity of the full CsCCD2 enzyme (Supplemental Fig. S2).

### The CsCCD2 intron-structure showed similarities with the genomic-structure of CCD1genes

**Fig. 3:**
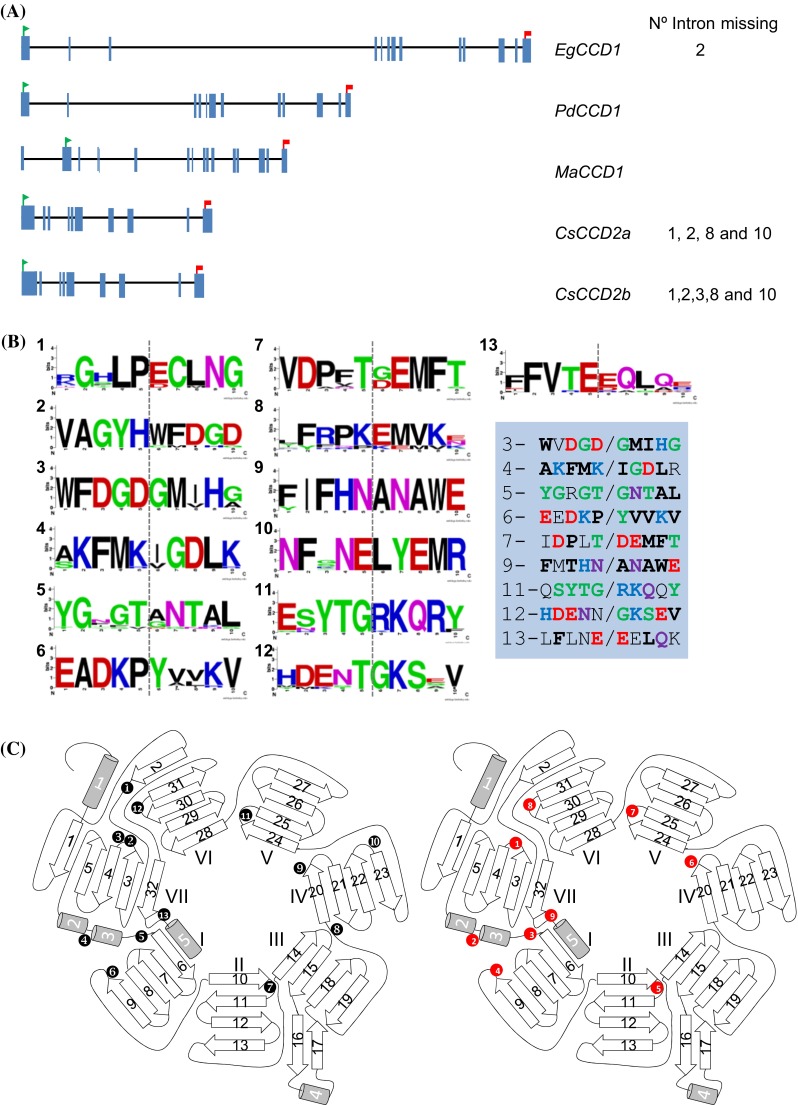
Intron-exons properties of *CsCCD2* and related *CCD1* genes and protein structure. **a** Genomic structure of *Elaeis guineensis CCD1*, *EgCCD1*; *Phoenix dactylifera CCD1*, *PdCCD1*; *Musa acuminata CCD1*, *MaCCD1* and *Crocus sativus* *CCD2* genes, *CsCCD2*. The *green flag* and *red flags* indicate the start and stop codons positions, respectively. **b** Amino acid consensus sequences around the insertion position of the conserved introns detected among the sequences listed in Supplemental Fig. S4. The *grey vertical bar* denotes the place where the intron is inserted in the nucleotide sequence. Intron number is indicated by *numbers*. In the *left*, in *blue shading* are presented the corresponding amino acid sequences in CsCCD2. Conserved amino acids are in *bold*. The *numbers* denote the intron inserted in the nucleotide sequence. **c** Schematic diagram of CCDs topology showing the seven-blade β-propeller as the basic motif. The location of the splice junctions mapped on the protein sequence are shown in the *left* as numbers with *black background* for the CCD1 sequences, and on the *right* as numbers with *red background* for the introns of CsCCD2a (where number *1* correspond with intron 3 in CCD1, number *2* with intron 4, number *3* with intron 5, number *4* with intron 6, number *5* with intron 7, number 6 with intron 9, number 7 with intron 11, number *8* with intron 12 and number *9* with intron 13)

The CCD2 enzymes are closely related to the CCD1 enzymes (Supplemental Fig. S3). Since the absence of other *CCD2* genomic sequences in the GenBank database, to further study the genomic structure of *CsCCD2*, the genomic clones were compared by Clustal with the *CCD1* genes from other plant species and were selected the ones with the highest identity score at the nucleotide level (*Phoenix dactylifera*, *PdCCD1* 79 %; *Musa acumuniata*, *MaCCD1* 77 %; *Elais guineensis*, *EgCCD1* 79 %) for intron/exon structure comparison (Fig. 3a, b). A distinct pattern of intron sizes emerged, and introns were observed in a total of 13 positions in the coding region. Introns were numbered according to placement starting with intron 1 closest to the start codon, and in all the observed introns, placement was conserved (Fig. 3a, b). In *CsCCD2a*, introns 1, 2, 8 and 10 were absent (Fig. 3a) and in *CsCCD2b* also intron 3 was missing. Intron sizes were compared among the selected *CCD1* and the *CsCCD2* sequences (Supplemental Table S1), and the unique relative conserved intron regarding size was intron 7. However, sequence comparison among the intron sequences did not revealed any identity and/or significant similarity with any coding or non-coding sequence.

The 5′ and 3′-splice sites sequences for the intronic *CsCCD2* sequences were compared with the consensus sequences of the *CCD1* genes from the monocot species used for intron position analysis (Fig. 3, Supplemental Fig. S3). The obtained consensus sequences (Table 2) were compared with the consensus sequences in plants (A/C)AG|GU(A/G)AGU and the UGCAG|G, at the donor and acceptor splice sites, respectively. The degree of conservation was high for both splice sites. By contrast, in the *CsCCD2* sequence the 5′-splice sites sequences were more conserved than the 3′-splice sites (Table 2).

**Table 2:** The 5′ and 3′-splice sites sequences for the intronic *CsCCD2* sequences, compared with the consensus sequences of the *CCD1* genes from the monocot species

Intron number	5′ (A/C)AG|gu(a/g)agu	3′ cag|G	Intron phase
3	GAUGguaaca	ugcaGAAU	1
4	GAAGguagau	gcagAUUG	0
5	GACUgguaac	auucGGUA	0
6	CCUUguaagu	caagAUGU	1
7	AACUgguaau	caacGAUG	2
9	UAAUgguuuc	uagcGCAA	0
11	ACUGgcaggu	aauuAGGA	0
12	AUGGguacua	aguaAAAU	1
13	UGAGagguua	uacuGAAG	0

Comparison of the intron/exon structure of these sequences with other monocot and dicot *CCD1* genomic sequences (Supplemental Fig. S3) allowed the verification of a consensus position for each intron on the amino acid sequence (Fig. 3b), which were also present in CsCCD2 (Fig. 3a). Interestingly, the introns appear in the loops linking elements of the secondary structure (Fig. 3b) rather than in either the alpha-helical or beta-sheet segments. Such intron pattern often appeared to correlate with junctions between domains or modules of protein structure as if the gene had been assembled at an early time from an exon-sized protogene precursor (de Souza et al. 1996). Further, the analysis of the positions of the intronic sequences in the CsCCD2 sequence showed that certain introns delineate the structural elements of the CsCCD2 enzyme tertiary structure (Fig. 3c). Intronic sequences 1 and 2 delineate the bladeVII; intronic sequences 3 and 4 delimitated blade I; intronic sequences 5 and 6 delimitated blades II and III; intronic sequences 6 and 7 delimitated blade IV; intronic sequences 7 and 8 delimitated blades V and VI; in the seven-blade β-propeller structure (Fig. 3c).

### Expression analysis of CsCCD2 in stigma tissue

The presence of the truncated clone encoding for an inactive enzyme, prompted us to evaluate its involvement in apocarotenoid regulation in saffron. To answer the question of whether the truncated gene was expressed, reverse transcriptase-PCR assays combined with RACE-PCR experiments were performed with RNA from yellow and orange stigmas using oligonucleotides to ensure the isolation of the complete *CsCCD2* cDNA (Supplemental Fig. S4). Two different populations differing in size were obtained after the amplification reactions, and the sequence of each population confirmed the expression of the truncated clone in both developmental stages. Both kind of clones showed in the 5′-UTR the consensus sequence A(−4)(A/G)(−3)(A/C)(−2)A(−1)ATGG(+4)C(+5)(U/G)(+6) for the plant genes (Nakagawa et al. 2008) and a 357 bp at the non-coding 3′ region excluding the poly(A) tail which starts 29 bp downstream from a putative polyadenylation signal, AATAAT.

**Fig. 4:**
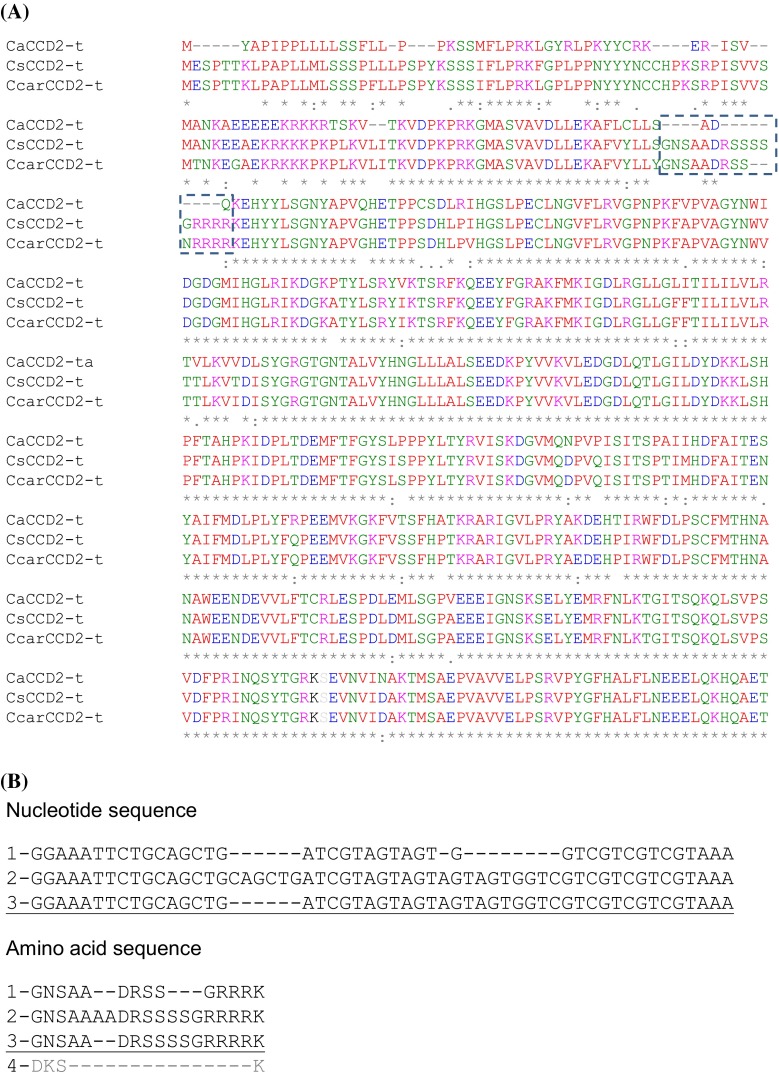
Alignment of amino acid sequences of CCD truncated proteins. **a** Amino acid sequences of CsCCD2 truncated (CsCCD2-t) homologues detected in *C. ancyrensis* (CaCCD2-t) and *C. cartwrightianus* (CcarCCD2-t). *Asterisk* indicates conserved amino acid residues. The *hatched box* denotes the deletion observed in CaCCD2-t and CcarCCD2-t. **b** Comparison at the nucleotide and amino acid level of the different 5′ regions of the *CsCCD2* genes amplified in the RACE-PCR experiment. Number *1*, *2* and *3* are all for *CsCCD2* variants, and number *4* for *CsCCD1*

To determine the possible functional implications of CsCCD2-t, we investigated the presence of *CsCCD2*-*t* homologous in other *Crocus* species, one of them closely related to *C. sativus*, *C. cartwrightianus* also flowering in autumn (Castillo et al. 2005), and the other one flowering in spring, *C. ancyrensis* (Rubio Moraga et al. 2013). The truncated form of *CCD2* was isolated from the stigmas of *C. ancyrensis* and *C. cartwrightianus* (Fig. 4a), suggesting a conserved function for such isoform in all the analyzed species. The main observed differences among the isolated sequences were found in the N-t region of the amino acid sequences (Fig. 4a). Interestingly, among the full length *CsCCD2* clones isolated in the RACE-PCR experiments, three different classes were isolated, which differed in the same 5′ region identified as different for the CCD2 truncated clones (Fig. 4a, b, Supplementary Fig. 4), and this region is absent in the CCD1 protein from *C. sativus* (amino acid sequence 4 [UniprotID Q84KG5], in Fig. 4b). This region does not correspond to any of the positions were introns are located in the genomic sequence (Fig. 3a).

### Promoter in silico analysis

Genome walker analyses allowed the isolation of a promoter region for *CsCCD2*. Using different primer combinations from the promoter and the coding sequence and performing sequence analysis on the amplified band, the promoter sequence corresponded to *CsCCD2b*. The promoter sequence isolated was 1575 bp long and was analysed using PLACE, RegSite Database of Plant Regulatory Elements and PlantCARE databases. Several putative regulatory elements were detected in these analyses (Table 3, Supplemental Table S2). The promoter showed several light-regulatory units (LRUs), *cis*-acting elements involved in heat stress and low temperature responsiveness (flowering in saffron is temperature-controlled), a wound response element and stress-*cis*-acting elements. In addition, three *cis*-acting elements involved circadian control were detected in the promoter region. The CAANNNNATC motif is conserved in the promoters of clock-controlled light-harvesting complex protein genes (Lhc) (Piechulla et al. 1998), the CCACA (ACCACAAAA) motif has been found in the promoters of dawn-phased genes (Covington et al. 2008) and the protein box element (PBX; ATGGGCC) confers midnight-phased expression on a luciferase reporter gene (Harmer and Kay 2005).

**Table 3:** Putative *cis*-elements identified in *CsCCD2b* promoter

Category	*Cis*-acting Element	Sequence	Position
Light	ACE	AAAACGTTTA	+416, +753, +638
	G-Box	CACGTT	+385
	G-box	ACACGTGGC	−409, −729
	GATA-motif	GATAGGA	−468
	GTGGC-motif	CATCGTGTGGC	−967, −1199
	Sp1	GGGCGG	−526
		CC(G/A)CCC	+1205
	I-box	CTCTTATGCT	+1081
		ATGATATGA	−276, +1142
Heat-stress response	HSE	AAAAAATTTC	+345, +608, −471
Low-temperature response	LTR	CCGAAA	+210, +635, −299
MYB binding site involved in drought-inducibility	MBS1	AAAAAAC	+413
ABA response	ABRE	GACACGTGGC	−968
Wound-response	WUN	AAATTTCCT	+611
Core promoter element	TATA-box	ATATAT	−109
Common *cis*-acting element in promoter and enhancer regions	CAAT-box	CAAT	−97, −248, −662, −918, −1092
Stress signal integration	ERRE	AGCCGCC	+837
Circadian regulation	Circadian	CAANNNNATC	−512
	CCACA	ACCACAAAA	+857
	PBX	ATGGGCC	+1362

Light response, wounding, ABA responsiveness and circadian regulatory elements were also detected in other stigma-specific gene related with the carotenoid metabolism in saffron (Ahrazem et al. 2010a). In order to identify other putative common regulatory elements in both promoter sequences, we compare the *CsCCD2b* promoter with the promoter of *CsLycB2a* using the Promoterwise program (McWilliam et al. 2013). Seven common sequences were identified (Table 4) that does not correspond to any of the previously identified *cis*-regulatory elements.

**Table 4:** Commom sequences identified in *CsCCD2b* and *CstLcyB2a* promoters using PromoterWise

Sequence	*CsCCD2*	*CstLcyB2a*
GTTAGGACTT	GTTAGGACTT(−1542)	GTTAGGACTT(44)
TGTCACATATTTTTAT	TGTCACATATATTTTAT(−137)	TGTCACATTTTTAT(301)
GGTTAAAAAAATT	GGTTAAAAAAAATT(−948)	GGTTAAAAATAATT(319)
ATTTTTTTAACCGA	ATTTTTTTTAACCCGA(936)	ATTTTTTATAAACCGA(370)
AAAAATAAAAAAA	AAAAATAAAAAAA(−469)	AAAAATAAAAAAA(524)
CCAAA TTTATACGT	CCAAACTTTATACGT(−1232)	CCAAAATTTATACGT(558)
AAT TTTAACCCA	AATGTTTAACCCA(−1038)	AATTTTAACCCA(723)

### CsCCD2 expression analysis for validation of identified *cis*-acting regulatory elements involved in CsCCD2 regulation

**Fig. 5:**
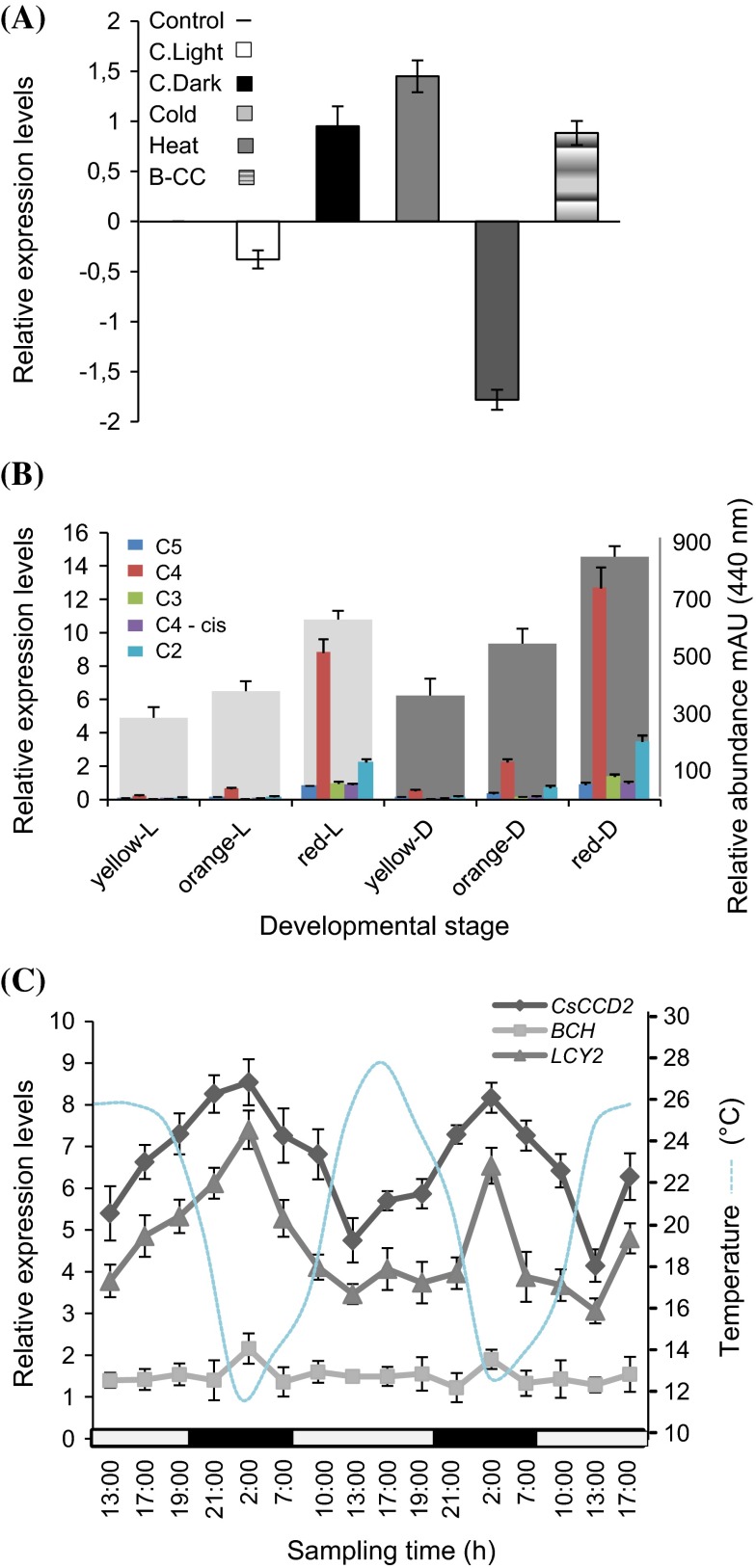
Validation of *CsCCD2* expression based on the elements identified by in silico promoter analysis by qRT-PCR. **a** Expression levels of *CsCCD2* under different light and temperature conditions. Represented values were obtained after subtracting the control value. **c** *Light* stands for continuous light and C. *Dark* stands for continuous dark. **b** Expression levels of *CsCCD2* in stigmas at different developmental stages (*yellow*, *orange*, and *red*) from plants growing in continuous dark (*yellow-D*, *orange-D* and *red-D*) or normal (*light*/*dark*) conditions (*yellow-L*, *orange-L* and *red-L*) are plotted on the *left y*-axe. Crocins levels (on the *right* axe) for each treatment and developmental stage are also indicated. C5, *trans*-crocin with 5 glucose molecules; C4, *trans*-crocin with 4 glucose molecules; C4-*cis*, *cis*-crocin with 4 glucose molecules; C3, *trans*-crocin with 3 glucose molecules; C2, *trans*-crocin with 2 glucose molecules crocins abundance on. **c** Expression data of *CsCCD2*, *CsBCH2* and *CsLycB2a* from plants growing in field. Expression levels are plotted on the *left* *y*-axe, and temperature on the *right*. *White box* on *x*-axe, *light* interval; *black box*, *dark* interval

The in silico analysis of the *CsCCD2b* promoter showed the presence of light-regulatory units (LRUs), and *cis*-acting elements involved in heat stress and low temperature responsiveness. Low temperature induced the expression of *CsCCD2* whereas high temperature repressed its expression (Fig. 5a). Similar results were observed for the effect of light on *CsCCD2* expression. Light repressed *CsCCD2* expression and dark conditions induced *CsCCD2* expression in the stigma tissue (Fig. 5a). In addition, when the stigma from plants growing in dark conditions were assayed for *CsCCD2* expression and crocin content, *CsCCD2* levels and crocin content were higher in the stigma derived from plants growing completely under dark conditions than in plants growing in normal light/dark conditions (Fig. 5b). Stigmas were also treated with β-cyclocitral (B-CC), produced in the earlier developmental stages of the stigma in the absence of light (Rubio et al. 2008), but in Arabidopsis it accumulates in leaves under high-light stress (Ramel et al. 2012). Under our conditions, β-cyclocitral induced the expression of *CsCCD2* similarly to the treatment in darkness (Fig. 5a).

Due to the involvement of light and temperature in the control of *CsCCD2* expression, we performed quantitative RT-PCR experiments to identify any patterns of *CsCCD2* transcript fluctuation over a normal ambient daily light/darkness period. *CsCCD2* expression displayed a clear rhythmic nature. The level of *CsCCD2* expression increased during the night period, and maximum *CsCCD2* transcript accumulation was detected at approximately at 2:00 a.m.; the level then declined an average of 1.7-fold during the light period (1:00 p.m.) (Fig. 5c). Similarly, we found such rhythmic expression pattern for the transcripts of two saffron genes encoding proteins involved in carotenoid biosynthesis in the stigma tissue of saffron; the β-ring carotene hydroxylase, *CsBCH1* gene (Castillo et al. 2005) and the lycopene-β-cyclase gene, *CstLcyB2a* (Ahrazem et al. 2010a) (Fig. 5c). *CstLcyB2a* expression levels reached its higher level during the night period (2:00 p.m.) and the level declined at least twofold during the light period (1:00 p.m.), while *CsBCH1* gene expression showed a 1.4-fold fluctuation between (2:00 a.m. and 1:00 p.m.).

### Identification of alternative splicing events in CsCCD2

**Fig. 6:**
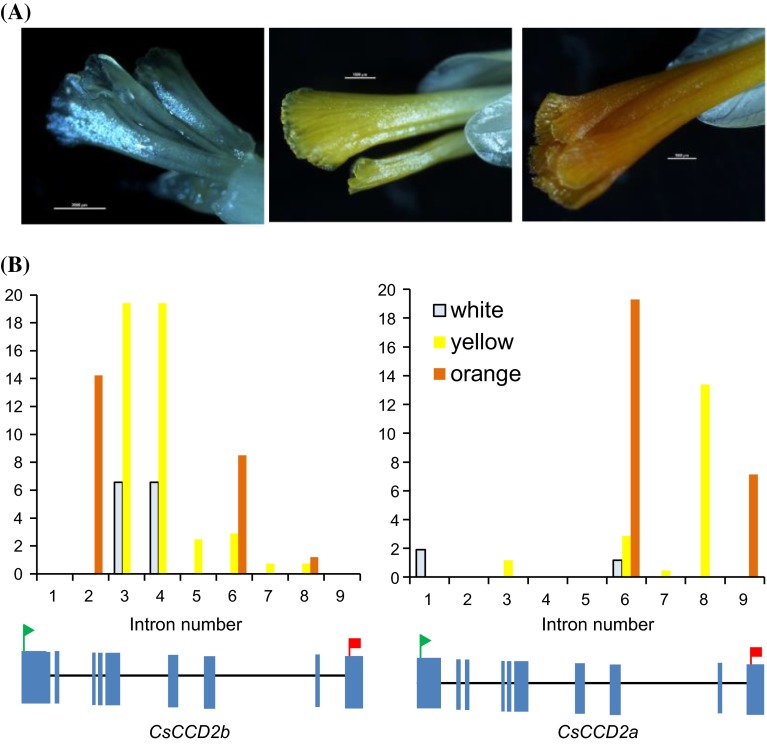
Splice variants of *CsCCD2* in three key developmental stages of saffron stigma. **a** Three developmental flower stages characterized by crocin accumulation in the stigma tissue. **b** Representation of the percentage of intron retention transcripts identified in the RNA-seq datasets from the stages presented in **a**

Post-transcriptional control of mRNA is a key regulatory mechanism that modulates plant responses to several environmental changes, including temperature and light fluctuations (Reddy et al. 2013). Using different primers combinations we observed the presence of longer and shorter products of *CsCCD2* compared with the expected size product in RT-PCR experiments using cDNA from yellow and orange stigmas (Supplemental Fig. S5). To explore the extent and conservation of alternative splicing (AS), if any, in *CsCCD2* genes, we evaluated in three saffron RNA-seq datasets (Supplemental Table S3) obtained from white, yellow and orange stigma (Fig. 6a) the presence of alternative splicing or intron retention in *CsCCD2* transcripts. In addition, the obtained data allowed us to get strand orientation information, which was used to identify the *CsCCD2* genomic sequences and their respective transcripts, as they were in different orientations. Transcripts with introns were identified in all the three datasets, but in comparison with *CsCCD2* transcripts without introns, they were more predominant in white (68.7 %) and yellow stages (30.2 %) and than in the orange stage (15.8 %) (Fig. 6b). Interestingly, intron variants follow a different behaviour depending on the gene from which they came from. Whereas intron 2, 3 and 4 were predominantly retained in *CsCCD2b*, introns 6, 8 and 9 were predominantly retained in *CsCCD2a*. Further, we analysed the recent published transcriptomes from several tissues from *C. sativus* (Jain et al. 2016) in order to determine the presence of intron retention in *CsCCD2* in those tissues were saffron apocarotenoids do not accumulated (Rubio-Moraga et al. 2010). In corm and stamens 100 % of the *CsCCD2* sequences contained introns, in leaves 91 % of the *CsCCD2* sequences contained introns, and in tepals 87 % of the detected *CsCCD2* sequences contained introns. Intronic sequences 2 and 3 were predominantly retained followed by intron 6 and 4.

Analyses of the putative protein sequences derived from the different intron retention events showed that intron 9 retention in *CsCCD2a* resulted in a protein 11 amino acids longer, where none of the key conserved residues for the catalytic activity of CCD proteins were affected (Supplemental Fig. S6). By contrast, the retention of all the other introns resulted in proteins depleted in structural motives from the seven-blade β-propeller; the number of blades is getting reduced as the intron retained is closer to the start codon (Supplemental Fig. S7).

## Discussion

The saffron *CCD2* gene, *CsCCD2*, has previously been shown to encode the enzyme responsible for crocetin production in saffron (Frusciante et al. 2014). *CsCCD2* expression is developmentally regulated and the enzyme localized in chromoplasts (Ahrazem et al. 2016), where crocetin is produced. Thereafter crocetin is glucosylated to crocins (Moraga et al. 2004), which are transported to the vacuoles, where accumulate (Grilli-Caiola and Canini 2004). This pathway remains largely uncharacterized, and major questions include the mechanisms that regulate *CsCCD2* expression and activity. In this study, we extended the structural and functional analysis on CsCCD2 to shed light on the regulation of this enzyme during the development of the stigma in saffron and its relation with other CCD enzymes. We found that the production of crocetin from the 7,8(7′,8′) cleavage of zeaxanthin, results from a evolutionary specialization from a CCD ancestor of the CCD1 sub-family. Further, to investigate the control of *CsCCD2* gene expression during the development of the stigma of saffron, we cloned a promoter fragment upstream of *CsCCD2* directly from *C. sativus* genomic DNA by PCR-genome walker. Several types of *cis*-regulatory elements were identified and some were found to be shared with the promoter region of the chromoplast-specific lycopene cyclase encoding gene *CsLycB2a* (Ahrazem et al. 2010a). The identification of light and temperature-responsive elements in both promoters has allowed us to determine the coordinated regulation of *CsLycB2a* and *CsCCD2* in saffron stigma.

### Intron loss in the CCD2 sequences

The carotenoid cleavage genes from animals and plants are characterized by the presence of several introns in their genomic sequences, with the exception of the *CCD4* family in plants, with a reduced intron number (Ahrazem et al. 2010b), suggesting the presence of intronic sequences in the ancestor of the carotenoid cleavage family (Ahrazem et al. 2010b). Phylogenetic relationships and the conservation of genomic organization throughout the CCD1 and CCD2 subfamilies provides a compelling argument for reconstructing the evolutionary history of CCD2 on the basis of a proposed pattern of intron loss from the common ancestral CCD from which CCD2 and CCD1 evolved. The *CCD1* sequences from the non-grass monocots contain 12–13 introns present in conserved positions. This intron number is also present in the genomic *CCD1* sequences from dicotyledonous species, suggesting the presence of at least 13 introns in the *CCD1* ancestor sequence. Two of the *CsCCD2* sequences analyzed have lost 4 and 5 intronic sequences. Interestingly, the first two introns were missing in both *CsCCD2* sequences. It has been shown that some introns are involved in the regulation of spatial or temporal expression of specific genes in different plants species, particularly, in the case of genes that are known to express constitutively, such as *PAT1*, *PhADF1*, *PRF1*, *2* and *3*; and *Ubi.U4*, first introns were required for their constitutive expression (Jeong et al. 2006; Mun et al. 2002; Plesse et al. 2001; Rose and Last 1997). In saffron, and in other plant species, *CCD1* is constitutively expressed in reproductive and vegetative tissues (Rubio et al. 2008), whereas *CsCCD2* expression is restricted to reproductive tissues and its expression is developmentally controlled (Rubio et al. 2008). The first CCD1 intronic sequences analysed were relatively long, although the biological significance of long introns is not certain at present, long introns may serve as binding sites for various regulatory elements (Duret 2001; Marais et al. 2005). The lost of the first intronic sequences in the *CsCCD2* sequence might allow a restricted specialization in terms of the specific expression pattern. Such regulation can be tissue-specific, controlled by developmental cues or modulated in response to external stimuli (Reddy et al. 2013).

We identify a *CsCCD2* genomic sequence without introns, which also lacks one exon. This sequence, *CsCCD2*-*t*, could be arisen by a retroposition event, since retroposition uses mature mRNA as the template instead of intron-containing pre-mRNA (Brosius 2003; Kaessmann et al. 2009). Interestingly, the *CsCCD2*-*t* gene is expressed in the stigmas of saffron but at lower levels than *CsCCD2* (Supplemental Fig. S4), although was as well developmentally regulated and was only detected in the early developmental stages of the stigma. A *CsCCD2*-*t* gene homolog was also detected in other two *Crocus* species that belong to different sections of the genus *Crocus*, section *Crocus* and section *Nudiscapus* which grouped 150 species of the genus, indicating its presence before the speciation process, and suggesting a functional role in the studied species. *CsCCD2*-*t* codifies for a protein of 480 amino acids, while CsCCD2 is 622 amino acids long. The CsCCD2-t is an inactive enzyme most probably due to the absence of important residues for catalytic activity. Truncated proteins may still perform part of their function and may act as dominant-negative regulators. Indeed, several recent studies provide evidence in support of such a dominant regulatory role for truncated proteins produced by splice variants in plants (Reddy et al. 2013). The Arabidopsis CCD1 enzyme has been suggested to act as a dimer (Schwartz et al. 2001), if this is also the case for CsCCD2 the presence of CsCCD2-t could be part of a regulatory mechanism for CsCCD2 activity.

### Splice variants in *CsCCD2*

The presence of several introns allowed the detection of different splice variants for the *CsCCD2* genes. These mRNA isoforms may differ in the untranslated regions (UTRs) with functional implications in transcript localization, stability, or translation. It has been estimated that close to 50 % of plant genes have alternative transcript isoforms (Filichkin et al. 2010) with retention of introns as the most frequent kind of alternative splicing (Staiger and Green 2011) and is prominent in stress-response pathways (Mastrangelo et al. 2012; Ner-Gaon et al. 2004). Recent mapping of eukaryotic transcriptomes and splicesomes using massively parallel RNA sequencing (RNA-seq) has revealed that the extent of alternative splicing has been considerably underestimated (Filichkin et al. 2010). Evidence also suggests that many pre-mRNAs undergo unproductive alternative splicing resulting in incorporation of in-frame premature termination codons. We have investigated alternative splicing in *CsCCD2* at three key developmental stages regarding crocetin biosynthesis and crocins accumulation (Moraga et al. 2009). Splice variants mainly accumulate in white and yellow stages, and we detected differences in the splice variants depending on the *CsCCD2* gene copy. In the *CsCCD2a* the preferred retained introns were the introns 6, 8 and 9; whereas in the *CsCCD2b* introns 2, 3 and 4 were the preferred retained ones. Perhaps the presence of intron 1 favours the recruitment of splicing factors for the more efficient splicing of introns 2, 3 and 4 in *CsCCD4a*. Further, the *CsCCD2* introns retained were not equally represented in the three analysed stages; introns 2, 6 and 9 were preferentially retained in transcripts identified in the orange stages, whereas introns 3, 4 and 8 were preferentially retained in the yellow stages. Certain splicing signals (i.e., *cis* elements) could reside in the introns or junction sites of *CsCCD2*. In accordance to this idea, *CsCCD2* splice-site sequences differ among them and from the consensus sequences obtained from the CCD1 monocot sequences. It has been suggested that weaker splice sites, the presence or absence of splicing regulators, RNA secondary structures, the exon/intron architecture, and the process of pre-mRNA synthesis itself are associated with intron retention (Hertel 2008). In addition, *cis*-regulatory elements are likely to play as well a crucial role in regulating intron retention by specific RNA-binding proteins and suggest a biological significance for this process in marking exons that are poised for alternative splicing (Sakabe and de Souza 2007). Alternative splicing has also been observed in *PSY*, suggested to be a key regulatory step in carotenoid biosynthesis (Cazzonelli and Pogson 2010; Fu et al. 2014; Rodriguez-Suarez et al. 2011). *PSY* alternative splicing in wheat results in the generation of four different transcripts, being only one functional, thereby titrating the level of functional PSY (Howitt et al. 2009). This can be the case for *CsCCD2*, the obtained data showed the presence of intron retention, which is much more evident in those tissues that do not accumulated crocins, where practically not functional *CsCCD2* transcripts without introns were detected.

### Promoter analysis

Transcriptional activation of gene expression seems to be the major form of regulation of carotenoid biosynthesis in many fruits and flowers (Fraser and Bramley 2004; Hirschberg 2001). Bio-informatics analyses of the obtained sequence identified putative TATA and CAAT boxes in the promoter. In addition, several light-response motifs were identified in the promoter, e.g. G-box, G-Box, Ace, I-Box, GATA-motif, Sp1 and GTGGC-motif, providing the basis for the regulation of apocarotenogenic gene expression according to day length and other cues involved in the control of flower development in saffron (Molina et al. 2005b). At least one circadian regulatory *cis*-element was also identified in the promoter of *CsCCD2* and two regulatory elements associated with phase-specific transcript accumulation.

A number of studies showed that light controls carotenoid biosynthesis in different species during fruit ripening, flower and root development (Fuentes et al. 2012; Kishimoto et al. 2004; Liu et al. 2004; Stange et al. 2008; Zhu et al. 2002). Moreover, *CCD1* transcript levels displayed circadian regulation in petunia and *Osmathus* flowers (Baldermann et al. 2010; Simkin et al. 2004) suggesting that carotenoid modification is also regulated by light. In addition, an in silico analysis with Genevestigator (www.genevestigator.com) indicated that the four *CCD* genes of *Arabidopsis* are not induced by light.

Other regulatory elements related with abiotic stress responses were identified in the promoter region such as a three HSE elements involved in heat stress responsiveness and three LTR elements involved in low-temperature responsiveness. While relative high temperatures are necessary for flower induction in saffron (during May–June), flower emergence and further stigma development in saffron required low temperature (15–17 °C) (September–October) (Molina et al. 2005a). *CsCCD2* expression is restricted to the stigma tissues (Rubio et al. 2008) and the presence of temperature response elements may help to coordinate *CsCCD2* expression and stigma development in saffron. While high temperatures repressed *CsCCD2* expression, *CsCCD2* was up-regulated by low temperatures, and in plants growing in the field the highest *CsCCD2* expression levels were reached when the lowest temperatures were measured (12–13 °C). In addition, an ABRE element, which is responsive to ABA is present in the *CsCCD2* promoter and ABA signalling plays a vital role in plant stress responses, including cold (Wang et al. 2003).

The integration of expression profiles and promoter sequences can help to identify common and putative functionally relevant *cis*-acting elements (Ma and Bohnert 2007). Comparative bio-informatics studies on promoter regions of carotenoid genes may elucidate common binding motifs involved in carotenoid formation (Fraser and Bramley 2004). We therefore compared the isolated *CsCCD2* promoter sequence with the *CsLycB2a* promoter (Ahrazem et al. 2010a) using the Plant-CARE and PLACE databases. Both promoters contained several light response elements, at least one ABRE element, and a circadian regulatory motif. Furthermore, both promoters shared several domains that could represent putative *cis*-acting elements controlling the coordinated expression of these genes in the early developmental stages of the stigma.

### Regulation of *CsCCD2* expression

We have observed that the steady-state transcript levels of *CsCCD2* are subjected to a daily rhythmic pattern of expression with higher expression levels during the night and at the time of lower temperatures. In addition, the same behaviour was observed for the chromoplast-specific genes *CsLycB2a* and *CsBCH1.* The light/dark regulation of carotenoid biosynthesis has been investigated in red pepper, where all transcript levels of genes involved in the carotenoid biosynthesis decreased under dark conditions (Simkin et al. 2003b) and in tomato leaves where the phytoene desaturase and ξ-carotene desaturase genes increased just prior to the light period (Simkin et al. 2003a). In citrus, the transcript levels of genes encoding enzymes involved in carotenoid biosynthesis are affected by the light quality (Kato et al. 2004; Zhang et al. 2012). The transcript abundance of carotenogenic genes in *Arabidopsis* is controlled by light, and also clock controlled, with genes showing a peak during the night period (Covington et al. 2008). However, in contrast to the dawn-phased transcript accumulation of carotenoid biosynthetic genes, a gene encoding a violaxanthin de-epoxidase in *Arabidopsis* leaves, showed the highest expression at subjective dusk (Covington et al. 2008). This antiphasic transcript accumulation pattern respect to the other carotenoid genes coincides well with the antagonistically activity of violaxanthin de-epoxidase, which acts recycling the carotenoid violaxanthin into compounds upstream of violaxanthin synthesis (Jahns et al. 2009). In all the analysed cases, the observed regulation of the carotenogenic genes is associated to the essential role that carotenoids play in the process of nonphotochemical quenching, which allows plants to quench excited chlorophyll and prevent oxidative damage under excessive light conditions. However, this is not the case for the biosynthesis of carotenoids in saffron stigma where carotenoid formation takes place in the absence of light (Moraga et al. 2009; Rubio et al. 2008), in the underground, as occurs in cassava (Henrique et al. 2007), sweetpotato (Teow et al. 2007) and carrots (Rodriguez-Concepcion and Stange 2013). In carrots, light prevents the differentiation of chromoplasts and modulates the expression of most carotenogenic genes while the absence of light contributes to the differentiation of chromoplasts and the accumulation of carotenoids. Our results showed that in fact darkness promotes higher accumulation of crocins and *CsCCD2* expression, while continuous light repressed *CsCCD2* expression. During the development of the stigma *CsCCD2* expression drops in the stigma of buds and flowers that have already emerged to the light (Frusciante et al. 2014; Rubio et al. 2008), the same is observed for crocins, which content has been increased as the stigma develops in the underground (Moraga et al. 2009), showing that flower development in saffron is coordinated with apocarotenoid biosynthesis in the stigma tissue, as observed for other *Crocus* species (Ahrazem et al. 2015a), suggesting a highly interconnected regulatory network that coordinates flower development and apocarotenoid biosynthesis. Therefore, the control of nuclear gene expression in response to the developmental state of plastids should be critical for the synthesis and accumulation of crocins in the chromoplast by the activity of *CsCCD2*. Based on the retrograde signalling concept in plastids, signals originating from the chromoplast might modulate the expression of the nuclear gene *CsCCD2*. Interestingly, in the promoter of nuclear genes associated with this retrograde signalling the ACGT motif, and the core of both the light-responsive G box (CACGTG) and the ABA response element (ABRE) are over-represented (Koussevitzky et al. 2007), and all these elements are present in the promoter of *CsCCD2*, that could be a clear target for the retrograde signal generated in the chromoplast that modulates *CsCCD2* expression.

**Fig. 7:**
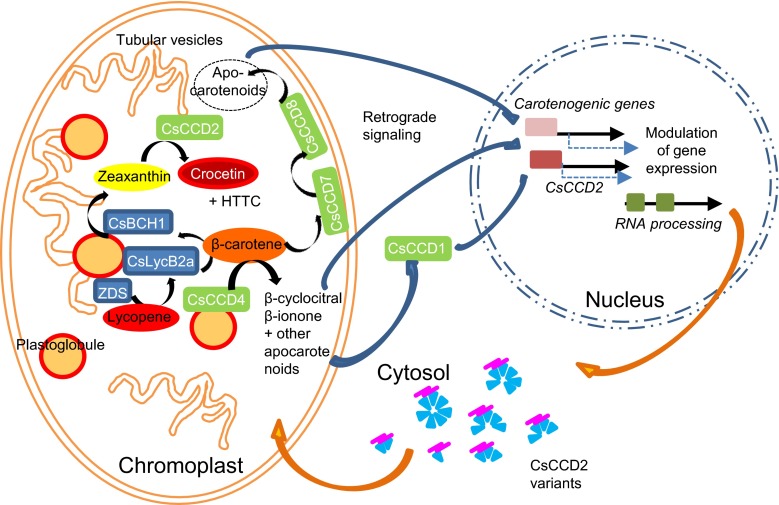
Schematic representation of the putative retrograde signalling pathway from the chromoplast to the nucleus for crocetin accumulation and chromoplast development. Different kinds of apocarotenoids are produced by CCDs in the chromoplast of saffron stigma in a developmental-dependent way. Many of these apocarotenoids could diffuse through the chromoplast membranes into the cytosol and act directly as signals for gene expression modulation or could be further modified by CCD1. The resulting apocarotenoids may act as the inducers of gene expression modulation of carotenogenic genes, *CsCCD2* and other gene targets related to chromoplast biogenesis and homeostasis during flower development in saffron

Metabolites are the most likely candidates for retrograde signalling molecules and three apocarotenoids including ABA and β-cyclocitral, are conceived as putative retrograde signals (Estavillo et al. 2012; Leister 2012). β-cyclocitral is produced in saffron stigma in early developmental stages, from yellow to red, following an accumulation pattern similar to *CsCCD2* expression (Moraga et al. 2009), and it could be considered as a putative signal molecule derived from the chromoplast responsible for *CsCCD2* activation (Fig. 7). In fact, *CsCCD2* expression was activated by β-cyclocitral, although the involvement of other apocarotenoids generated by the activity of the different CCDs expressed in the saffron stigma cannot be excluded from this model (Ahrazem et al. 2010b; Rubio-Moraga et al. 2014a, b) (Fig. 7). Furthermore, because these apocarotenoids can affect major developmental processes (Avendano-Vazquez et al. 2014; Barbier et al. 2015) they can function as well as feedback signals responding to the state of organelle development. In fact, changes in organellar function are known to result in corresponding changes of at least some metabolite pool sizes, which define distinct metabolic states or metabolite signatures (Pfannschmidt 2010).

The control mechanism of gene regulation by these signals still mainly unknown (Estavillo et al. 2012), but recently it has been shown that a chloroplast retrograde signal regulate nuclear alternative splicing (Petrillo et al. 2014). Alternatively spliced genes are involved in a broad range of plant functions, such as signal transduction, growth and development, responses to stresses, the circadian clock, flowering time, metabolism and physiology (Kazan 2003). In addition, unproductive alternative splicing allows rapid and steep adjustments in mRNA levels, allowing the synchronization of physiological processes with periodic environmental changes (Filichkin et al. 2015; Filichkin and Mockler 2012; Sanchez et al. 2011). Certainly such a mechanism could be extended to other retrograde signalling pathways explaining the alternative splicing observed in *CsCCD2*.

## Electronic supplementary material

Below is the link to the electronic supplementary material.

Supplementary material 1 (PPTX 2773 kb)

Supplementary material 2 (DOCX 16 kb)

Supplementary material 3 (DOCX 56 kb)
