# Supplementary material for: Intron retention and rhythmic diel pattern regulation of carotenoid cleavage dioxygenase 2 during crocetin biosynthesis in saffron
Source: Plant Mol Biol. 2016 Apr 12;91:355–74. doi: 10.1007/s11103-016-0473-8 (PMC4884571; doi:10.1007/s11103-016-0473-8)
Supplement: Supplementary file 1 — Supplementary material 1 (PPTX 2773 kb) [file 11103_2016_473_MOESM1_ESM.pptx]

## Slide 1
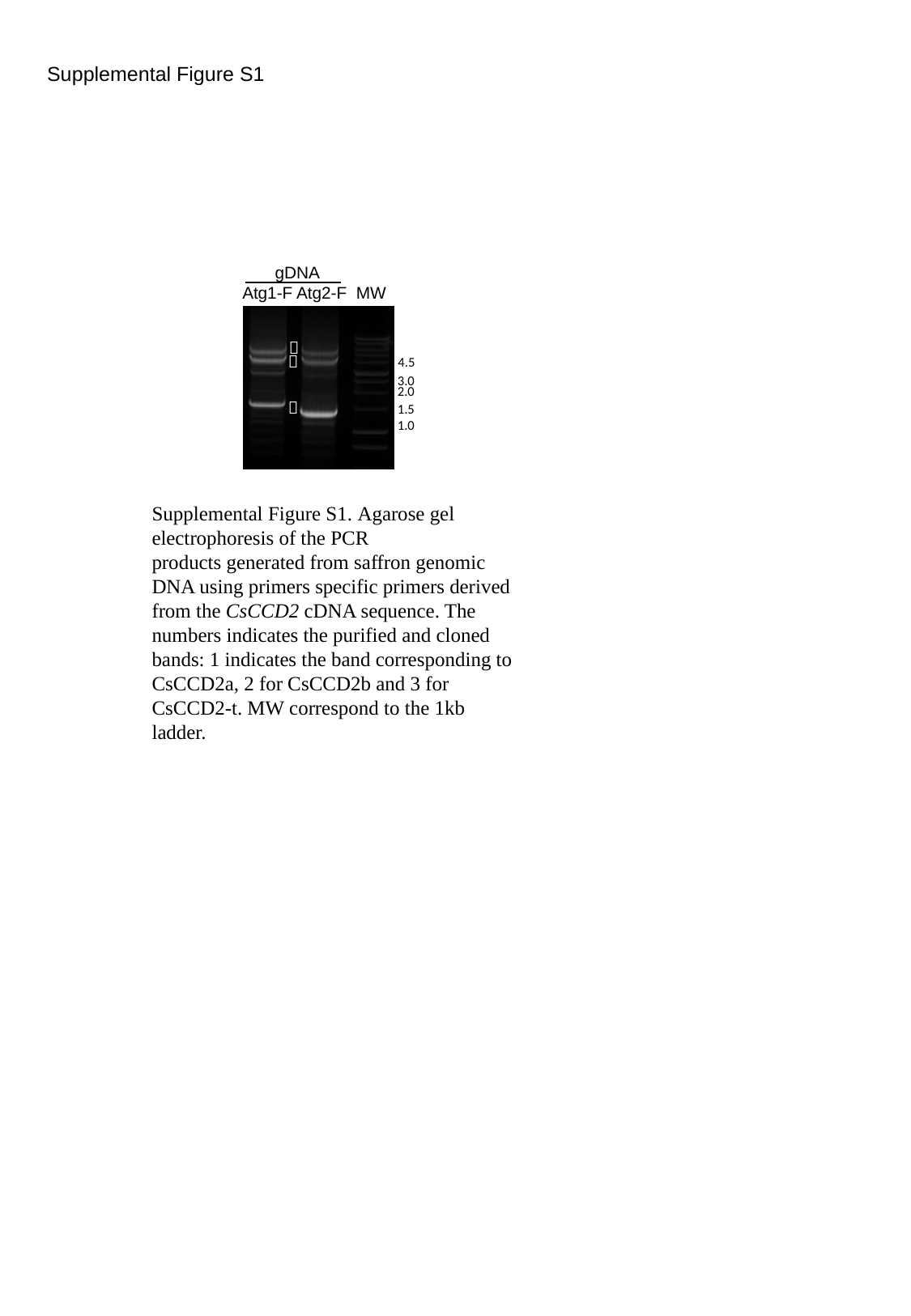

Supplemental Figure S1
 gDNA
 Atg1-F Atg2-F MW

4.5


3.0
2.0
1.5
1.0
Supplemental Figure S1. Agarose gel electrophoresis of the PCR
products generated from saffron genomic DNA using primers specific primers derived from the CsCCD2 cDNA sequence. The numbers indicates the purified and cloned bands: 1 indicates the band corresponding to CsCCD2a, 2 for CsCCD2b and 3 for CsCCD2-t. MW correspond to the 1kb ladder.

## Slide 2
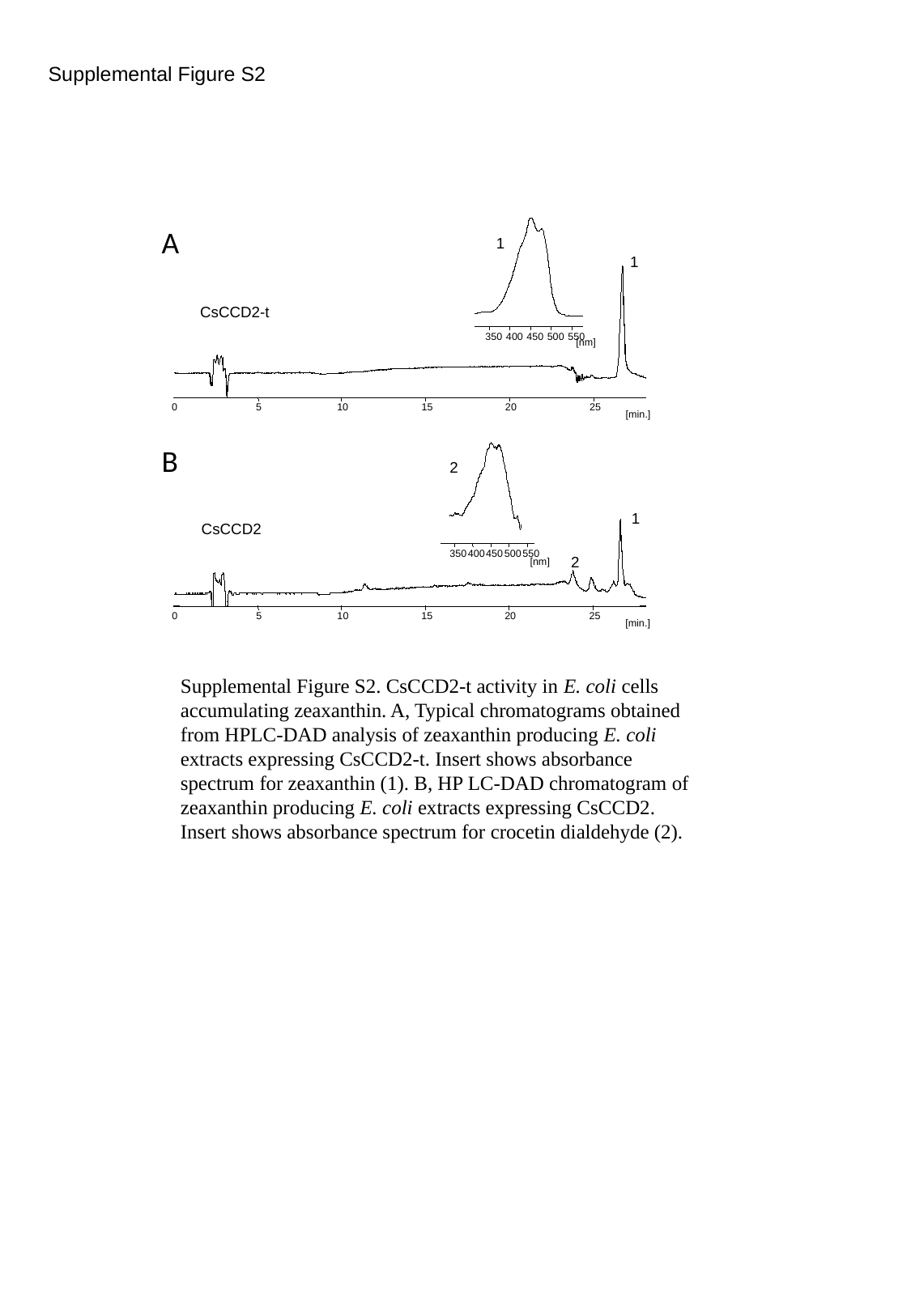

Supplemental Figure S2
A
B
350
400
450
500
550
[nm]
1
1
CsCCD2-t
0
5
10
15
20
25
[min.]
350
400
450
500
550
[nm]
2
1
CsCCD2
0
5
10
15
20
25
[min.]
2
Supplemental Figure S2. CsCCD2-t activity in E. coli cells accumulating zeaxanthin. A, Typical chromatograms obtained from HPLC-DAD analysis of zeaxanthin producing E. coli extracts expressing CsCCD2-t. Insert shows absorbance spectrum for zeaxanthin (1). B, HP LC-DAD chromatogram of zeaxanthin producing E. coli extracts expressing CsCCD2. Insert shows absorbance spectrum for crocetin dialdehyde (2).

## Slide 3
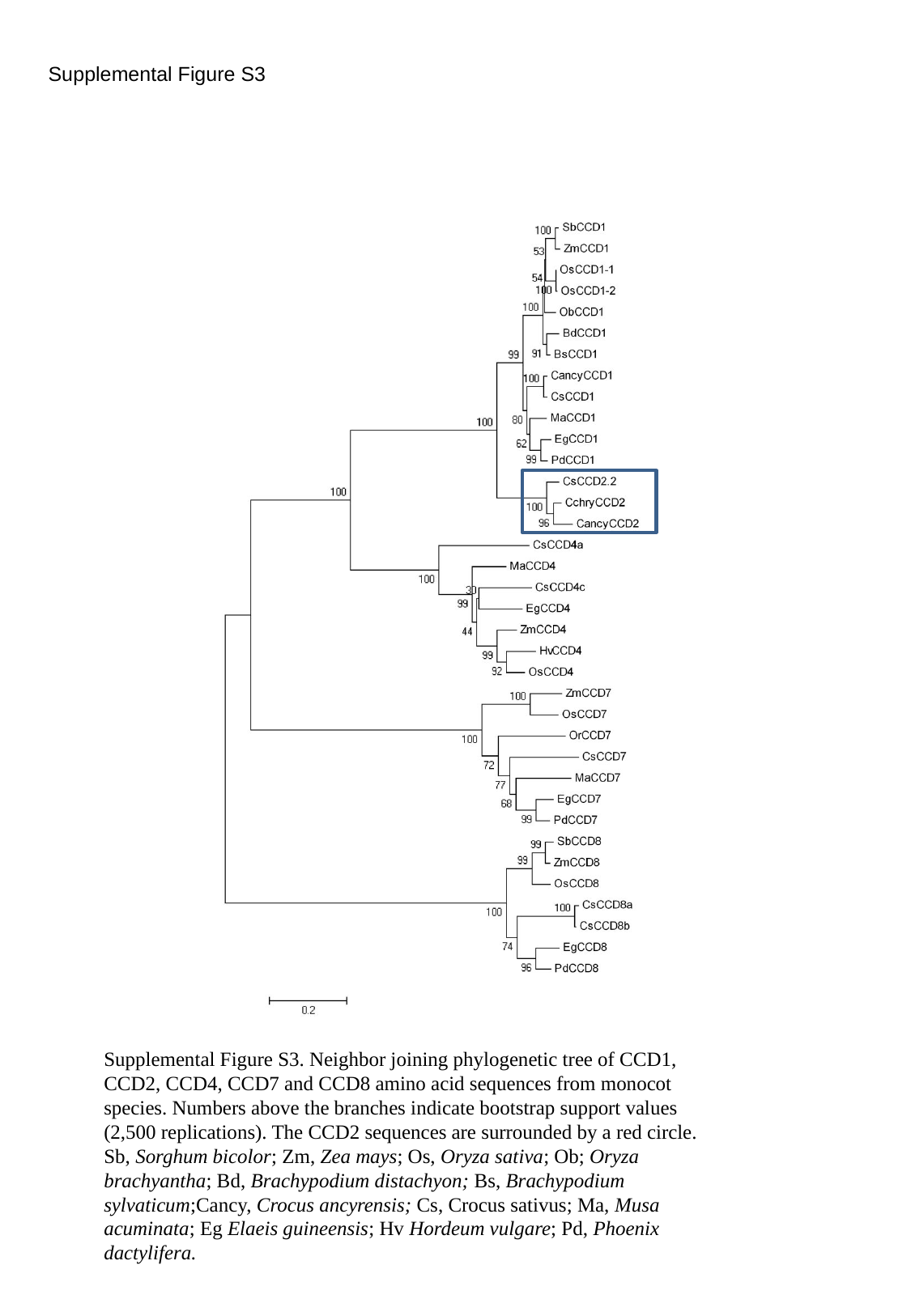

Supplemental Figure S3
Supplemental Figure S3. Neighbor joining phylogenetic tree of CCD1, CCD2, CCD4, CCD7 and CCD8 amino acid sequences from monocot species. Numbers above the branches indicate bootstrap support values (2,500 replications). The CCD2 sequences are surrounded by a red circle. Sb, Sorghum bicolor; Zm, Zea mays; Os, Oryza sativa; Ob; Oryza brachyantha; Bd, Brachypodium distachyon; Bs, Brachypodium sylvaticum;Cancy, Crocus ancyrensis; Cs, Crocus sativus; Ma, Musa acuminata; Eg Elaeis guineensis; Hv Hordeum vulgare; Pd, Phoenix dactylifera.

## Slide 4
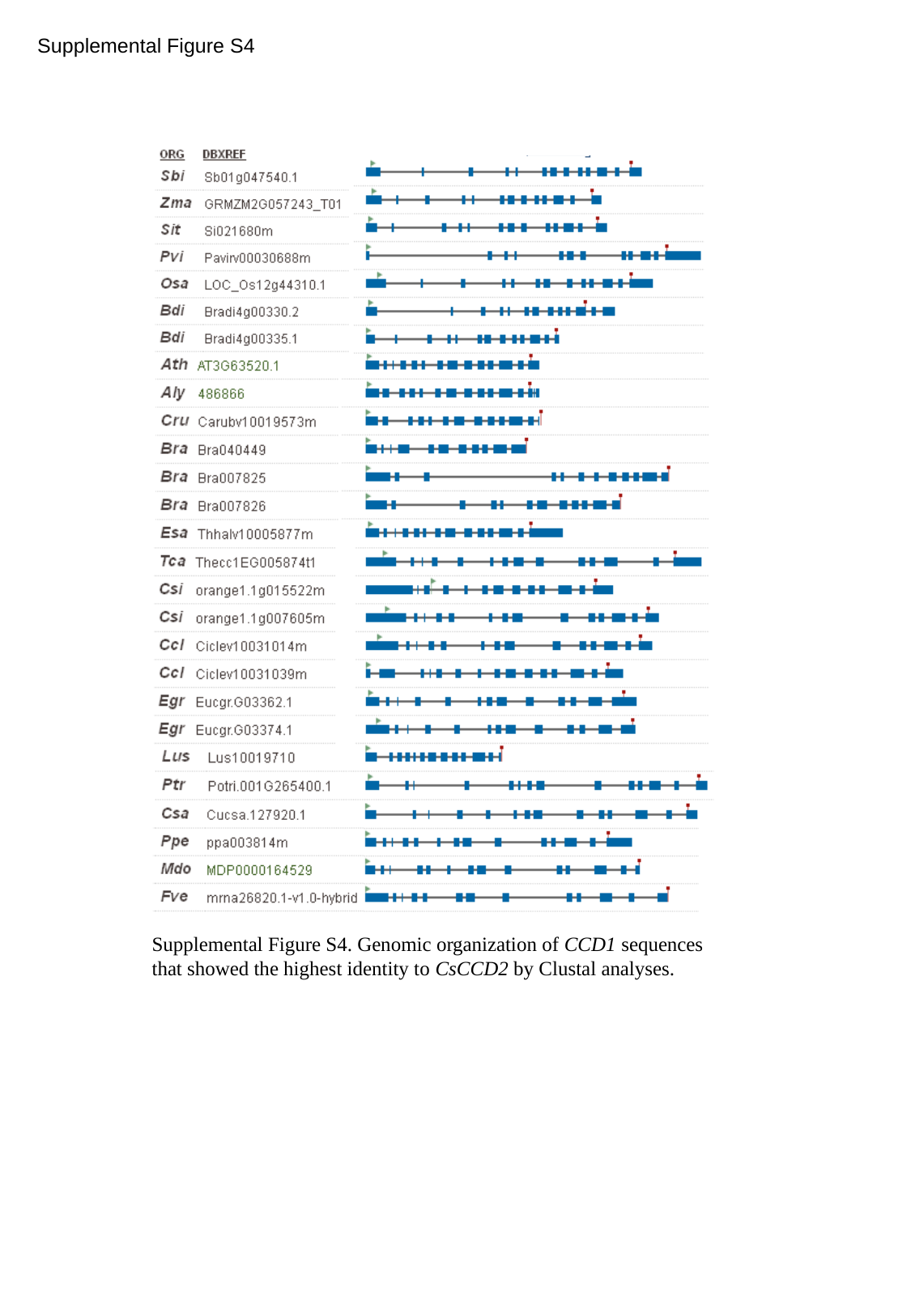

Supplemental Figure S4
Supplemental Figure S4. Genomic organization of CCD1 sequences that showed the highest identity to CsCCD2 by Clustal analyses.

## Slide 5
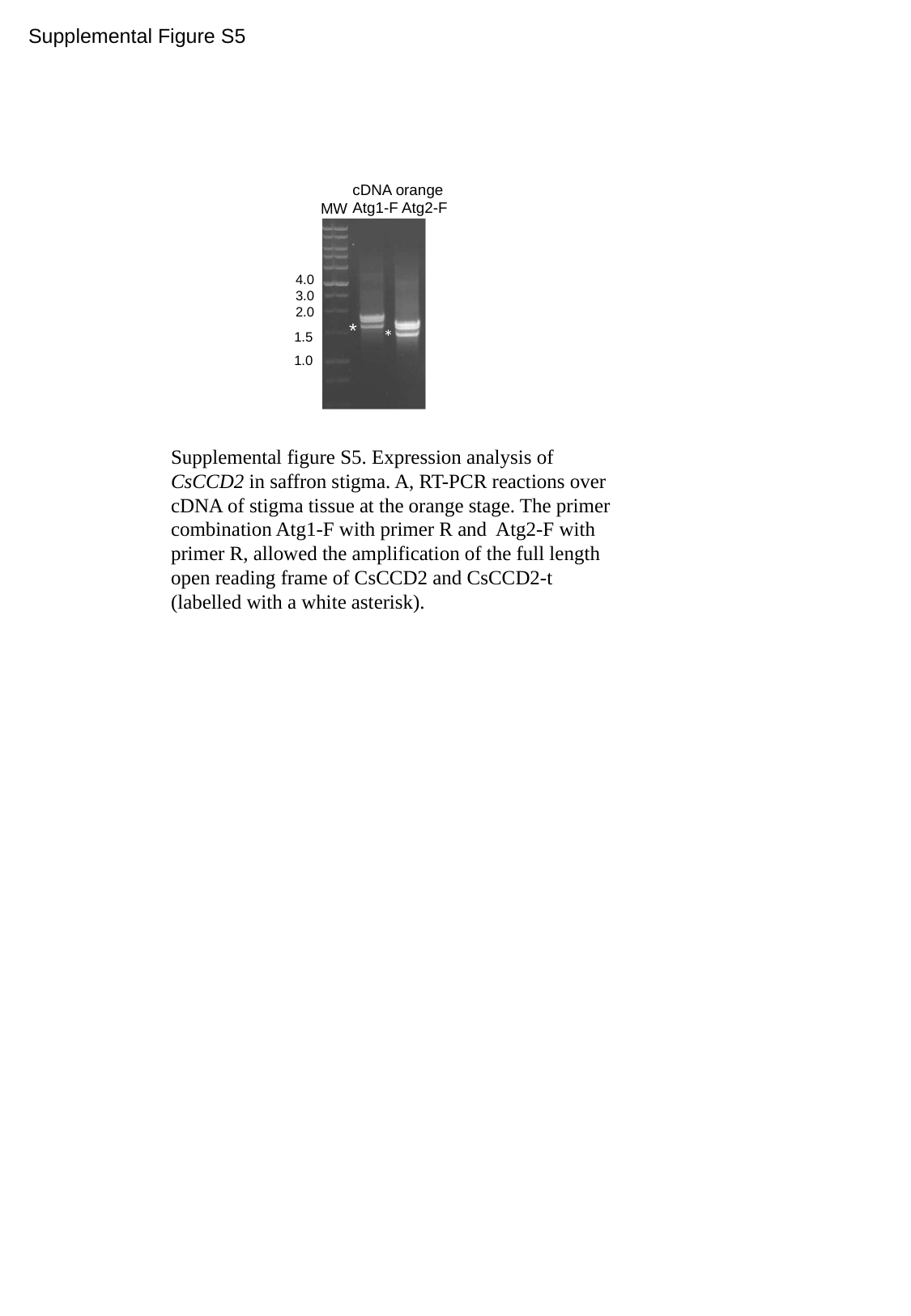

Supplemental Figure S5
cDNA orange
Atg1-F Atg2-F
MW
4.0
3.0
2.0
*
1.5
1.0
*
Supplemental figure S5. Expression analysis of CsCCD2 in saffron stigma. A, RT-PCR reactions over cDNA of stigma tissue at the orange stage. The primer combination Atg1-F with primer R and Atg2-F with primer R, allowed the amplification of the full length open reading frame of CsCCD2 and CsCCD2-t (labelled with a white asterisk).

## Slide 6
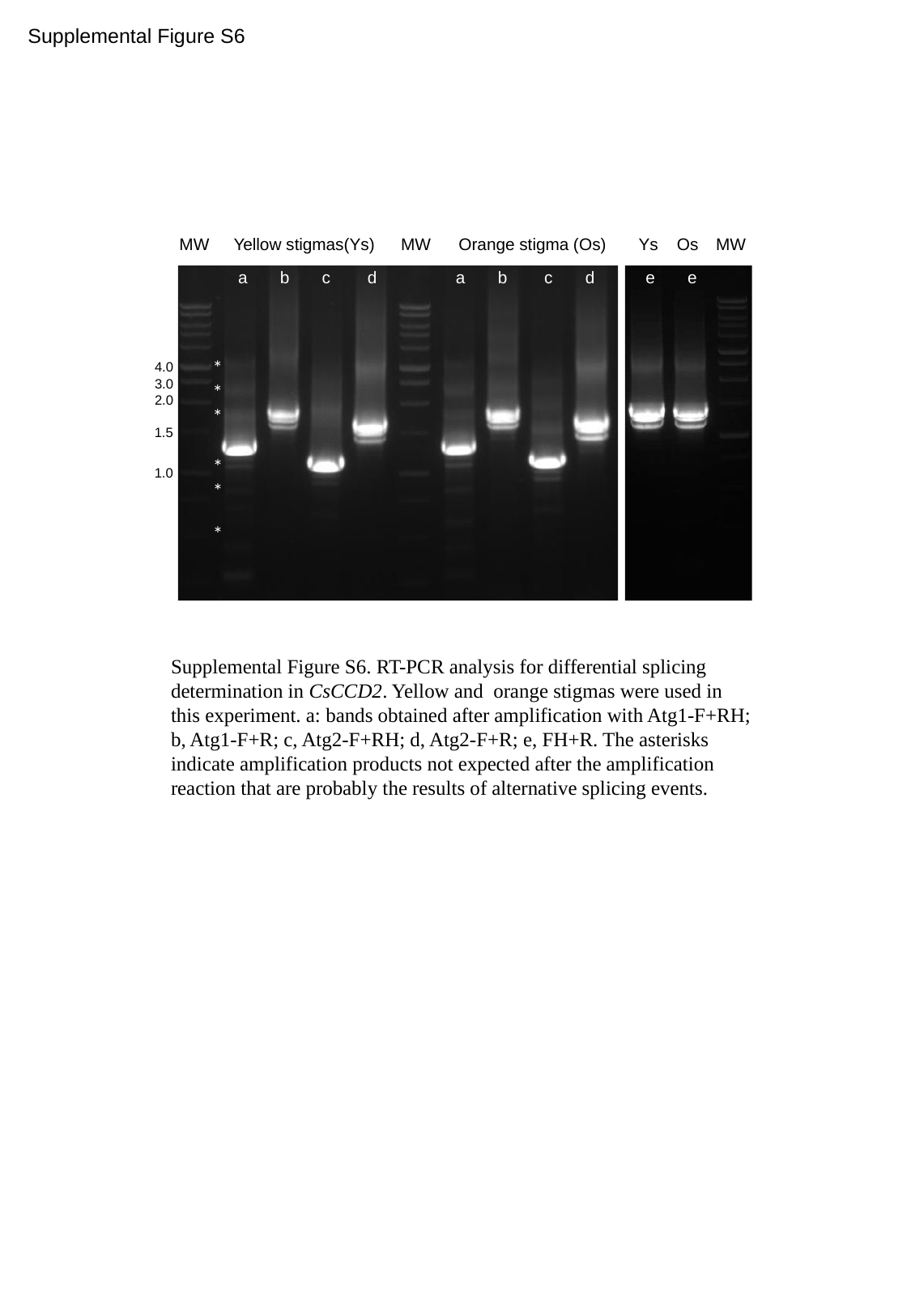

Supplemental Figure S6
Yellow stigmas(Ys) Orange stigma (Os) Ys Os
MW
MW
MW
a b c d a b c d e e
*
*
*
4.0
3.0
2.0
1.5
1.0
*
*
*
Supplemental Figure S6. RT-PCR analysis for differential splicing determination in CsCCD2. Yellow and orange stigmas were used in this experiment. a: bands obtained after amplification with Atg1-F+RH; b, Atg1-F+R; c, Atg2-F+RH; d, Atg2-F+R; e, FH+R. The asterisks indicate amplification products not expected after the amplification reaction that are probably the results of alternative splicing events.

## Slide 7
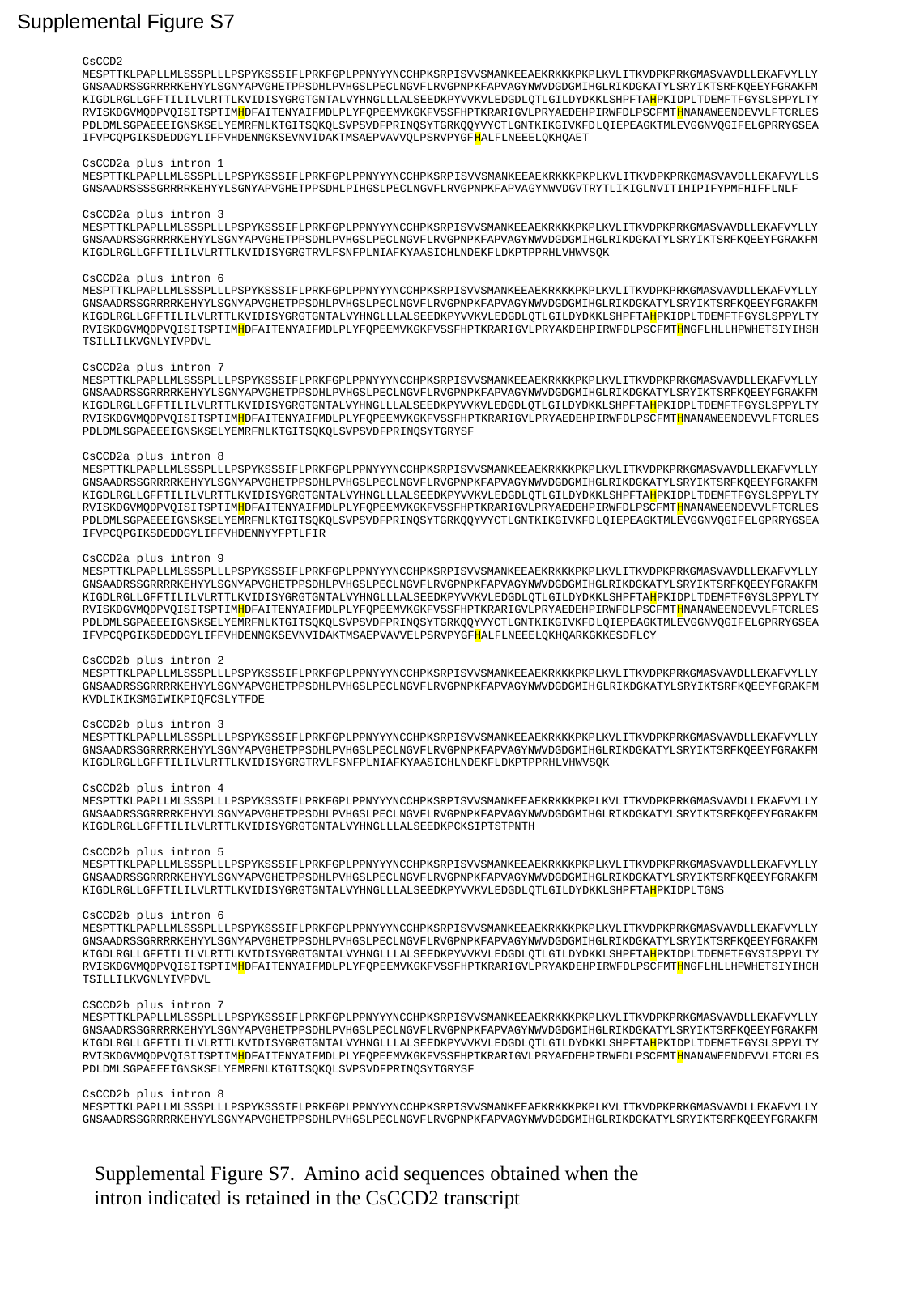

Supplemental Figure S7
Supplemental Figure S7. Amino acid sequences obtained when the intron indicated is retained in the CsCCD2 transcript

## Slide 8
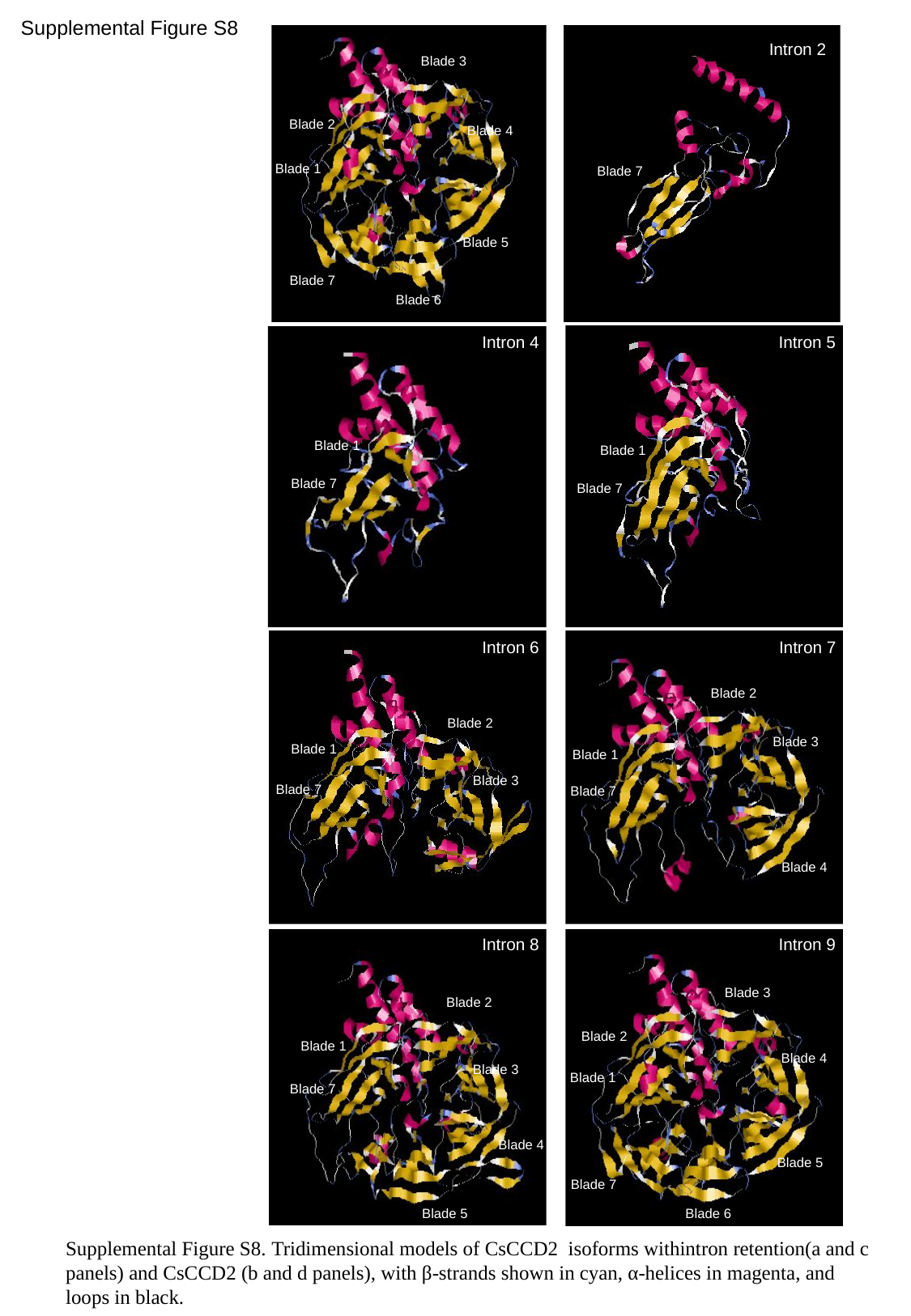

Supplemental Figure S8
Blade 3
Blade 2
Blade 4
Blade 1
Blade 5
Blade 7
Blade 6
Intron 2
Blade 7
Intron 5
Intron 4
Blade 1
Blade 1
Blade 7
Blade 7
Intron 6
Intron 7
Blade 2
Blade 2
Blade 3
Blade 1
Blade 1
Blade 3
Blade 7
Blade 7
Blade 4
Intron 8
Intron 9
Blade 3
Blade 2
Blade 2
Blade 1
Blade 4
Blade 3
Blade 1
Blade 7
Blade 4
Blade 5
Blade 7
Blade 5
Blade 6
Supplemental Figure S8. Tridimensional models of CsCCD2 isoforms withintron retention(a and c panels) and CsCCD2 (b and d panels), with β-strands shown in cyan, α-helices in magenta, and loops in black.
