# Supplementary material for: Intron retention and rhythmic diel pattern regulation of carotenoid cleavage dioxygenase 2 during crocetin biosynthesis in saffron
Source: Plant Mol Biol. 2016 Apr 12;91:355–74. doi: 10.1007/s11103-016-0473-8 (PMC4884571; doi:10.1007/s11103-016-0473-8)
Supplement: Supplementary file 2 — Supplementary material 2 (DOCX 16 kb) [file 11103_2016_473_MOESM2_ESM.docx]

Supplemental Table S1. Intron number and size in *Phoenix datilifera*, *PdCCD1*; *Musa acumuniata*, *MaCCD1*; *Elais guineensis* *EgCCD1* and *Crocus sativus*, *CsCCD2*.

| Intron number | *PhCCD1* | *EgCCD1* | *MaCCD1* | *CsCCD2b* | *CsCCD2a* |
| --- | --- | --- | --- | --- | --- |
| 1 | 1555 | 1613 | 433 | - | - |
| 2 | 1733 | - | 702 | - | - |
| 3 | 5238 | 1622 | 1519 | - | 325 |
| 4 | 164 | 10971 | 2082 | 122 | 122 |
| 5 | 175 | 170 | 105 | 725 | 725 |
| 6 | 282 | 289 | 432 | 87 | 87 |
| 7 | 84 | 84 | 81 | 77 | 77 |
| 8 | 169 | 166 | 175 | - | - |
| 9 | 2276 | 2400 | 795 | 1095 | 1126 |
| 10 | 80 | 78 | 70 | - | - |
| 11 | 1400 | 1417 | 828 | 550 | 550 |
| 12 | 769 | 617 | 118 | 2190 | 2396 |
| 13 | 221 | 208 | 591 | 527 | 324 |

Supplemental Table S3

| **Sample** | **Total Bases** | **Read Count** | **N (%)** | **GC (%)** | **Q20 (%)** | **Q30 (%)** |
| --- | --- | --- | --- | --- | --- | --- |
| white_RNA | 5.750.991.308 | 56.940.508 | 0,004 | 55,13 | 92,50 | 85,69 |
| yellow_RNA | 6.414.337.896 | 63.508.296 | 0,004 | 55 | 92,46 | 85,57 |
| orange_RNA | 6.137.247.146 | 60.825.323 | 0,004 | 54 | 92,36 | 85,54 |
