# Supplementary material for: Intron retention and rhythmic diel pattern regulation of carotenoid cleavage dioxygenase 2 during crocetin biosynthesis in saffron
Source: Plant Mol Biol. 2016 Apr 12;91:355–74. doi: 10.1007/s11103-016-0473-8 (PMC4884571; doi:10.1007/s11103-016-0473-8)
Supplement: Supplementary file 3 — Supplementary material 3 (DOCX 56 kb) [file 11103_2016_473_MOESM3_ESM.docx]

Supplemental Table S2. *In silico* analysis of *CsCCD2* promoter.

| **Factor** | **Site** | **Strand** | **Seq** | **Species** | **Source** |
| --- | --- | --- | --- | --- | --- |
| [AGL3](http://plantpan.mbc.nctu.edu.tw/tf_id_search.php?tfid=%20P$AGL3_01%20%20%20%20%20%20%20%20%20%20%20%20%20&lib=combine&source=TRANSFAC) | 221 | - | tattagaacTATGGgccc | Arabidopsis | TRANSFAC |
| [AGL3](http://plantpan.mbc.nctu.edu.tw/tf_id_search.php?tfid=%20P$AGL3_01%20%20%20%20%20%20%20%20%20%20%20%20%20&lib=combine&source=TRANSFAC) | 396 | - | ctaatggaaTATGGcccc | Arabidopsis | TRANSFAC |
| [AGL3](http://plantpan.mbc.nctu.edu.tw/tf_id_search.php?tfid=%20P$AGL3_01%20%20%20%20%20%20%20%20%20%20%20%20%20&lib=combine&source=TRANSFAC) | 585 | - | tatcattagTATGGtgaa | Arabidopsis | TRANSFAC |
| [AGL3](http://plantpan.mbc.nctu.edu.tw/tf_id_search.php?tfid=%20P$AGL3_01%20%20%20%20%20%20%20%20%20%20%20%20%20&lib=combine&source=TRANSFAC) | 1017 | - | gtgtgaaaaTATGGgtta | Arabidopsis | TRANSFAC |
| [AGL3](http://plantpan.mbc.nctu.edu.tw/tf_id_search.php?tfid=%20P$AGL3_01%20%20%20%20%20%20%20%20%20%20%20%20%20&lib=combine&source=TRANSFAC) | 1358 | + | aaatCCATAggtgatcat | Arabidopsis | TRANSFAC |
| [AG](http://plantpan.mbc.nctu.edu.tw/tf_id_search.php?tfid=%20P$AG_01%20%20%20%20%20%20%20%20%20%20%20%20%20%20%20&lib=combine&source=TRANSFAC) | 248 | - | tcaaaaataTTTGGgaaa | Arabidopsis | TRANSFAC |
| [AG](http://plantpan.mbc.nctu.edu.tw/tf_id_search.php?tfid=%20P$AG_01%20%20%20%20%20%20%20%20%20%20%20%20%20%20%20&lib=combine&source=TRANSFAC) | 285 | + | tagtCCAAAcagcaaaag | Arabidopsis | TRANSFAC |
| [AG](http://plantpan.mbc.nctu.edu.tw/tf_id_search.php?tfid=%20P$AG_01%20%20%20%20%20%20%20%20%20%20%20%20%20%20%20&lib=combine&source=TRANSFAC) | 410 | - | cccccaattTTTGGgatc | Arabidopsis | TRANSFAC |
| [AG](http://plantpan.mbc.nctu.edu.tw/tf_id_search.php?tfid=%20P$AG_01%20%20%20%20%20%20%20%20%20%20%20%20%20%20%20&lib=combine&source=TRANSFAC) | 743 | + | aatgCCAAAagatttgac | Arabidopsis | TRANSFAC |
| [AG](http://plantpan.mbc.nctu.edu.tw/tf_id_search.php?tfid=%20P$AG_01%20%20%20%20%20%20%20%20%20%20%20%20%20%20%20&lib=combine&source=TRANSFAC) | 816 | - | gtcaaatctTTTGGtgct | Arabidopsis | TRANSFAC |
| [AG](http://plantpan.mbc.nctu.edu.tw/tf_id_search.php?tfid=%20P$AG_01%20%20%20%20%20%20%20%20%20%20%20%20%20%20%20&lib=combine&source=TRANSFAC) | 871 | + | ccgaCCAAAaaaacgtta | Arabidopsis | TRANSFAC |
| [AG](http://plantpan.mbc.nctu.edu.tw/tf_id_search.php?tfid=%20P$AG_01%20%20%20%20%20%20%20%20%20%20%20%20%20%20%20&lib=combine&source=TRANSFAC) | 1083 | - | taacattttTTTGGtccc | Arabidopsis | TRANSFAC |
| [AG](http://plantpan.mbc.nctu.edu.tw/tf_id_search.php?tfid=%20P$AG_01%20%20%20%20%20%20%20%20%20%20%20%20%20%20%20&lib=combine&source=TRANSFAC) | 1221 | - | cgtataaagTTTGGcatt | Arabidopsis | TRANSFAC |
| [ANT](http://plantpan.mbc.nctu.edu.tw/tf_id_search.php?tfid=%20P$ANT_01%20%20%20%20%20%20%20%20%20%20%20%20%20%20&lib=combine&source=TRANSFAC) | 229 | + | ctatgggCCCGAag | Arabidopsis | TRANSFAC |
| [ANT](http://plantpan.mbc.nctu.edu.tw/tf_id_search.php?tfid=%20P$ANT_01%20%20%20%20%20%20%20%20%20%20%20%20%20%20&lib=combine&source=TRANSFAC) | 763 | - | ttTCGGGtttcgat | Arabidopsis | TRANSFAC |
| [ANT](http://plantpan.mbc.nctu.edu.tw/tf_id_search.php?tfid=%20P$ANT_01%20%20%20%20%20%20%20%20%20%20%20%20%20%20&lib=combine&source=TRANSFAC) | 942 | + | tttttaaCCCGAtg | Arabidopsis | TRANSFAC |
| [ANT](http://plantpan.mbc.nctu.edu.tw/tf_id_search.php?tfid=%20P$ANT_01%20%20%20%20%20%20%20%20%20%20%20%20%20%20&lib=combine&source=TRANSFAC) | 1091 | + | ttttggtCCCGAaa | Arabidopsis | TRANSFAC |
| [Athb-1](http://plantpan.mbc.nctu.edu.tw/tf_id_search.php?tfid=%20P$ATHB1_01%20%20%20%20%20%20%20%20%20%20%20%20&lib=combine&source=TRANSFAC) | 212 | + | aatacATTATatta | Arabidopsis | TRANSFAC |
| [Athb-1](http://plantpan.mbc.nctu.edu.tw/tf_id_search.php?tfid=%20P$ATHB1_01%20%20%20%20%20%20%20%20%20%20%20%20&lib=combine&source=TRANSFAC) | 239 | + | gaaggATTATcaaa | Arabidopsis | TRANSFAC |
| [Athb-1](http://plantpan.mbc.nctu.edu.tw/tf_id_search.php?tfid=%20P$ATHB1_01%20%20%20%20%20%20%20%20%20%20%20%20&lib=combine&source=TRANSFAC) | 347 | + | tgaagATTATagac | Arabidopsis | TRANSFAC |
| [Athb-1](http://plantpan.mbc.nctu.edu.tw/tf_id_search.php?tfid=%20P$ATHB1_01%20%20%20%20%20%20%20%20%20%20%20%20&lib=combine&source=TRANSFAC) | 705 | - | gtaaATAATtaccg | Arabidopsis | TRANSFAC |
| [Athb-1](http://plantpan.mbc.nctu.edu.tw/tf_id_search.php?tfid=%20P$ATHB1_01%20%20%20%20%20%20%20%20%20%20%20%20&lib=combine&source=TRANSFAC) | 1152 | + | catgtATTATttaa | Arabidopsis | TRANSFAC |
| [Athb-1](http://plantpan.mbc.nctu.edu.tw/tf_id_search.php?tfid=%20P$ATHB1_01%20%20%20%20%20%20%20%20%20%20%20%20&lib=combine&source=TRANSFAC) | 1504 | + | taaatATTATaaac | Arabidopsis | TRANSFAC |
| [Athb-1](http://plantpan.mbc.nctu.edu.tw/tf_id_search.php?tfid=%20P$ATHB1_01%20%20%20%20%20%20%20%20%20%20%20%20&lib=combine&source=TRANSFAC) | 1576 | + | tcactATTATtctt | Arabidopsis | TRANSFAC |
| [ATHB-5](http://plantpan.mbc.nctu.edu.tw/tf_id_search.php?tfid=%20P$ATHB5_01%20%20%20%20%20%20%20%20%20%20%20%20&lib=combine&source=TRANSFAC) | 68 | - | aAATAAagt | Arabidopsis | TRANSFAC |
| [ATHB-5](http://plantpan.mbc.nctu.edu.tw/tf_id_search.php?tfid=%20P$ATHB5_01%20%20%20%20%20%20%20%20%20%20%20%20&lib=combine&source=TRANSFAC) | 158 | - | aAATAAaaa | Arabidopsis | TRANSFAC |
| [ATHB-5](http://plantpan.mbc.nctu.edu.tw/tf_id_search.php?tfid=%20P$ATHB5_01%20%20%20%20%20%20%20%20%20%20%20%20&lib=combine&source=TRANSFAC) | 461 | + | tttTTATTt | Arabidopsis | TRANSFAC |
| [ATHB-5](http://plantpan.mbc.nctu.edu.tw/tf_id_search.php?tfid=%20P$ATHB5_01%20%20%20%20%20%20%20%20%20%20%20%20&lib=combine&source=TRANSFAC) | 502 | + | ttcTTATTg | Arabidopsis | TRANSFAC |
| [ATHB-5](http://plantpan.mbc.nctu.edu.tw/tf_id_search.php?tfid=%20P$ATHB5_01%20%20%20%20%20%20%20%20%20%20%20%20&lib=combine&source=TRANSFAC) | 707 | - | aAATAAtta | Arabidopsis | TRANSFAC |
| [ATHB-5](http://plantpan.mbc.nctu.edu.tw/tf_id_search.php?tfid=%20P$ATHB5_01%20%20%20%20%20%20%20%20%20%20%20%20&lib=combine&source=TRANSFAC) | 1009 | - | aAATAAcag | Arabidopsis | TRANSFAC |
| [ATHB-5](http://plantpan.mbc.nctu.edu.tw/tf_id_search.php?tfid=%20P$ATHB5_01%20%20%20%20%20%20%20%20%20%20%20%20&lib=combine&source=TRANSFAC) | 1142 | - | aAATAAata | Arabidopsis | TRANSFAC |
| [ATHB-5](http://plantpan.mbc.nctu.edu.tw/tf_id_search.php?tfid=%20P$ATHB5_01%20%20%20%20%20%20%20%20%20%20%20%20&lib=combine&source=TRANSFAC) | 1146 | - | aAATAAcat | Arabidopsis | TRANSFAC |
| [ATHB-5](http://plantpan.mbc.nctu.edu.tw/tf_id_search.php?tfid=%20P$ATHB5_01%20%20%20%20%20%20%20%20%20%20%20%20&lib=combine&source=TRANSFAC) | 1155 | + | gtaTTATTt | Arabidopsis | TRANSFAC |
| [ATHB-5](http://plantpan.mbc.nctu.edu.tw/tf_id_search.php?tfid=%20P$ATHB5_01%20%20%20%20%20%20%20%20%20%20%20%20&lib=combine&source=TRANSFAC) | 1427 | - | aAATAAcgt | Arabidopsis | TRANSFAC |
| [ATHB-5](http://plantpan.mbc.nctu.edu.tw/tf_id_search.php?tfid=%20P$ATHB5_01%20%20%20%20%20%20%20%20%20%20%20%20&lib=combine&source=TRANSFAC) | 1495 | + | ggtTTATTc | Arabidopsis | TRANSFAC |
| [ATHB-5](http://plantpan.mbc.nctu.edu.tw/tf_id_search.php?tfid=%20P$ATHB5_01%20%20%20%20%20%20%20%20%20%20%20%20&lib=combine&source=TRANSFAC) | 1579 | + | ctaTTATTc | Arabidopsis | TRANSFAC |
| [ATHB-9](http://plantpan.mbc.nctu.edu.tw/tf_id_search.php?tfid=%20P$ATHB9_01%20%20%20%20%20%20%20%20%20%20%20%20&lib=combine&source=TRANSFAC) | 77 | + | gatagctATGATaaatggc | Arabidopsis | TRANSFAC |
| [ATHB-9](http://plantpan.mbc.nctu.edu.tw/tf_id_search.php?tfid=%20P$ATHB9_01%20%20%20%20%20%20%20%20%20%20%20%20&lib=combine&source=TRANSFAC) | 574 | - | aacatgaATCATatcatta | Arabidopsis | TRANSFAC |
| [ATHB-9](http://plantpan.mbc.nctu.edu.tw/tf_id_search.php?tfid=%20P$ATHB9_01%20%20%20%20%20%20%20%20%20%20%20%20&lib=combine&source=TRANSFAC) | 579 | - | gaatcatATCATtagtatg | Arabidopsis | TRANSFAC |
| [ATHB-9](http://plantpan.mbc.nctu.edu.tw/tf_id_search.php?tfid=%20P$ATHB9_01%20%20%20%20%20%20%20%20%20%20%20%20&lib=combine&source=TRANSFAC) | 768 | - | ggtttcgATCATatatctt | Arabidopsis | TRANSFAC |
| [ATHB-9](http://plantpan.mbc.nctu.edu.tw/tf_id_search.php?tfid=%20P$ATHB9_01%20%20%20%20%20%20%20%20%20%20%20%20&lib=combine&source=TRANSFAC) | 1046 | - | ttttttcATCATttgccgg | Arabidopsis | TRANSFAC |
| [ATHB-9](http://plantpan.mbc.nctu.edu.tw/tf_id_search.php?tfid=%20P$ATHB9_01%20%20%20%20%20%20%20%20%20%20%20%20&lib=combine&source=TRANSFAC) | 1364 | - | ataggtgATCATaggtact | Arabidopsis | TRANSFAC |
| [ATHB-9](http://plantpan.mbc.nctu.edu.tw/tf_id_search.php?tfid=%20P$ATHB9_01%20%20%20%20%20%20%20%20%20%20%20%20&lib=combine&source=TRANSFAC) | 1450 | + | ccaatgcATGATatgagtt | Arabidopsis | TRANSFAC |
| [CDC5](http://plantpan.mbc.nctu.edu.tw/tf_id_search.php?tfid=%20P$CDC5_01%20%20%20%20%20%20%20%20%20%20%20%20%20&lib=combine&source=TRANSFAC) | 334 | + | tttTCAGCaaa | Arabidopsis | TRANSFAC |
| [CDC5](http://plantpan.mbc.nctu.edu.tw/tf_id_search.php?tfid=%20P$CDC5_01%20%20%20%20%20%20%20%20%20%20%20%20%20&lib=combine&source=TRANSFAC) | 342 | - | aaaGCTGAaga | Arabidopsis | TRANSFAC |
| [CDC5](http://plantpan.mbc.nctu.edu.tw/tf_id_search.php?tfid=%20P$CDC5_01%20%20%20%20%20%20%20%20%20%20%20%20%20&lib=combine&source=TRANSFAC) | 828 | - | ggtGCTGAccc | Arabidopsis | TRANSFAC |
| [PIF3](http://plantpan.mbc.nctu.edu.tw/tf_id_search.php?tfid=%20P$PIF3_01%20%20%20%20%20%20%20%20%20%20%20%20%20&lib=combine&source=TRANSFAC) | 694 | - | ttgcctaACGTGtaaata | Arabidopsis | TRANSFAC |
| [RAV1](http://plantpan.mbc.nctu.edu.tw/tf_id_search.php?tfid=%20P$RAV1_01%20%20%20%20%20%20%20%20%20%20%20%20%20&lib=combine&source=TRANSFAC) | 149 | + | tacCAACAaaaa | Arabidopsis | TRANSFAC |
| [RAV1](http://plantpan.mbc.nctu.edu.tw/tf_id_search.php?tfid=%20P$RAV1_01%20%20%20%20%20%20%20%20%20%20%20%20%20&lib=combine&source=TRANSFAC) | 986 | - | aagtTGTTGcga | Arabidopsis | TRANSFAC |
| [GAmyb](http://plantpan.mbc.nctu.edu.tw/tf_id_search.php?tfid=%20P$GAMYB_01%20%20%20%20%20%20%20%20%20%20%20%20&lib=combine&source=TRANSFAC) | 376 | - | ttgGGTTG | Barley | TRANSFAC |
| [GAmyb](http://plantpan.mbc.nctu.edu.tw/tf_id_search.php?tfid=%20P$GAMYB_01%20%20%20%20%20%20%20%20%20%20%20%20&lib=combine&source=TRANSFAC) | 390 | + | CAACCcct | Barley | TRANSFAC |
| [GAmyb](http://plantpan.mbc.nctu.edu.tw/tf_id_search.php?tfid=%20P$GAMYB_01%20%20%20%20%20%20%20%20%20%20%20%20&lib=combine&source=TRANSFAC) | 848 | + | CAACCgac | Barley | TRANSFAC |
| [Dof1](http://plantpan.mbc.nctu.edu.tw/tf_id_search.php?tfid=%20P$DOF1_01%20%20%20%20%20%20%20%20%20%20%20%20%20&lib=combine&source=TRANSFAC) | 68 | + | aaaTAAAGtga | Maize | TRANSFAC |
| [Dof1](http://plantpan.mbc.nctu.edu.tw/tf_id_search.php?tfid=%20P$DOF1_01%20%20%20%20%20%20%20%20%20%20%20%20%20&lib=combine&source=TRANSFAC) | 483 | + | atcTAAAGtaa | Maize | TRANSFAC |
| [Dof1](http://plantpan.mbc.nctu.edu.tw/tf_id_search.php?tfid=%20P$DOF1_01%20%20%20%20%20%20%20%20%20%20%20%20%20&lib=combine&source=TRANSFAC) | 1222 | + | gtaTAAAGttt | Maize | TRANSFAC |
| [Dof2](http://plantpan.mbc.nctu.edu.tw/tf_id_search.php?tfid=%20P$DOF2_01%20%20%20%20%20%20%20%20%20%20%20%20%20&lib=combine&source=TRANSFAC) | 160 | + | ataaAAAGCta | Maize | TRANSFAC |
| [Dof2](http://plantpan.mbc.nctu.edu.tw/tf_id_search.php?tfid=%20P$DOF2_01%20%20%20%20%20%20%20%20%20%20%20%20%20&lib=combine&source=TRANSFAC) | 338 | + | cagcAAAGCtg | Maize | TRANSFAC |
| [Dof2](http://plantpan.mbc.nctu.edu.tw/tf_id_search.php?tfid=%20P$DOF2_01%20%20%20%20%20%20%20%20%20%20%20%20%20&lib=combine&source=TRANSFAC) | 1466 | + | gttaAAAGCgt | Maize | TRANSFAC |
| [Dof3](http://plantpan.mbc.nctu.edu.tw/tf_id_search.php?tfid=%20P$DOF3_01%20%20%20%20%20%20%20%20%20%20%20%20%20&lib=combine&source=TRANSFAC) | 160 | + | ataaAAAGCta | Maize | TRANSFAC |
| [Dof3](http://plantpan.mbc.nctu.edu.tw/tf_id_search.php?tfid=%20P$DOF3_01%20%20%20%20%20%20%20%20%20%20%20%20%20&lib=combine&source=TRANSFAC) | 338 | + | cagcAAAGCtg | Maize | TRANSFAC |
| [Dof3](http://plantpan.mbc.nctu.edu.tw/tf_id_search.php?tfid=%20P$DOF3_01%20%20%20%20%20%20%20%20%20%20%20%20%20&lib=combine&source=TRANSFAC) | 1466 | + | gttaAAAGCgt | Maize | TRANSFAC |
| [PBF](http://plantpan.mbc.nctu.edu.tw/tf_id_search.php?tfid=%20P$PBF_01%20%20%20%20%20%20%20%20%20%20%20%20%20%20&lib=combine&source=TRANSFAC) | 160 | + | ataAAAAGcta | Maize | TRANSFAC |
| [PBF](http://plantpan.mbc.nctu.edu.tw/tf_id_search.php?tfid=%20P$PBF_01%20%20%20%20%20%20%20%20%20%20%20%20%20%20&lib=combine&source=TRANSFAC) | 295 | + | agcAAAAGtga | Maize | TRANSFAC |
| [PBF](http://plantpan.mbc.nctu.edu.tw/tf_id_search.php?tfid=%20P$PBF_01%20%20%20%20%20%20%20%20%20%20%20%20%20%20&lib=combine&source=TRANSFAC) | 308 | + | agaAAAAGtga | Maize | TRANSFAC |
| [PBF](http://plantpan.mbc.nctu.edu.tw/tf_id_search.php?tfid=%20P$PBF_01%20%20%20%20%20%20%20%20%20%20%20%20%20%20&lib=combine&source=TRANSFAC) | 496 | - | cttCTTTTctt | Maize | TRANSFAC |
| [PBF](http://plantpan.mbc.nctu.edu.tw/tf_id_search.php?tfid=%20P$PBF_01%20%20%20%20%20%20%20%20%20%20%20%20%20%20&lib=combine&source=TRANSFAC) | 543 | - | ttaCTTTTtaa | Maize | TRANSFAC |
| [PBF](http://plantpan.mbc.nctu.edu.tw/tf_id_search.php?tfid=%20P$PBF_01%20%20%20%20%20%20%20%20%20%20%20%20%20%20&lib=combine&source=TRANSFAC) | 613 | - | gtaCTTTTgta | Maize | TRANSFAC |
| [PBF](http://plantpan.mbc.nctu.edu.tw/tf_id_search.php?tfid=%20P$PBF_01%20%20%20%20%20%20%20%20%20%20%20%20%20%20&lib=combine&source=TRANSFAC) | 746 | + | gccAAAAGatt | Maize | TRANSFAC |
| [PBF](http://plantpan.mbc.nctu.edu.tw/tf_id_search.php?tfid=%20P$PBF_01%20%20%20%20%20%20%20%20%20%20%20%20%20%20&lib=combine&source=TRANSFAC) | 781 | - | tatCTTTTgac | Maize | TRANSFAC |
| [PBF](http://plantpan.mbc.nctu.edu.tw/tf_id_search.php?tfid=%20P$PBF_01%20%20%20%20%20%20%20%20%20%20%20%20%20%20&lib=combine&source=TRANSFAC) | 820 | - | aatCTTTTggt | Maize | TRANSFAC |
| [PBF](http://plantpan.mbc.nctu.edu.tw/tf_id_search.php?tfid=%20P$PBF_01%20%20%20%20%20%20%20%20%20%20%20%20%20%20&lib=combine&source=TRANSFAC) | 892 | - | tttCTTTTgaa | Maize | TRANSFAC |
| [PBF](http://plantpan.mbc.nctu.edu.tw/tf_id_search.php?tfid=%20P$PBF_01%20%20%20%20%20%20%20%20%20%20%20%20%20%20&lib=combine&source=TRANSFAC) | 1103 | - | aaaCTTTTatc | Maize | TRANSFAC |
| [PBF](http://plantpan.mbc.nctu.edu.tw/tf_id_search.php?tfid=%20P$PBF_01%20%20%20%20%20%20%20%20%20%20%20%20%20%20&lib=combine&source=TRANSFAC) | 1252 | - | caaCTTTTtag | Maize | TRANSFAC |
| [PBF](http://plantpan.mbc.nctu.edu.tw/tf_id_search.php?tfid=%20P$PBF_01%20%20%20%20%20%20%20%20%20%20%20%20%20%20&lib=combine&source=TRANSFAC) | 1439 | - | agtCTTTTact | Maize | TRANSFAC |
| [PBF](http://plantpan.mbc.nctu.edu.tw/tf_id_search.php?tfid=%20P$PBF_01%20%20%20%20%20%20%20%20%20%20%20%20%20%20&lib=combine&source=TRANSFAC) | 1466 | + | gttAAAAGcgt | Maize | TRANSFAC |
| [P](http://plantpan.mbc.nctu.edu.tw/tf_id_search.php?tfid=%20P$P_01%20%20%20%20%20%20%20%20%20%20%20%20%20%20%20%20&lib=combine&source=TRANSFAC) | 60 | - | tgGGTAGaa | Maize | TRANSFAC |
| [P](http://plantpan.mbc.nctu.edu.tw/tf_id_search.php?tfid=%20P$P_01%20%20%20%20%20%20%20%20%20%20%20%20%20%20%20%20&lib=combine&source=TRANSFAC) | 1383 | + | ccCTACCta | Maize | TRANSFAC |
| [MYB.Ph3](http://plantpan.mbc.nctu.edu.tw/tf_id_search.php?tfid=%20P$MYBPH3_01%20%20%20%20%20%20%20%20%20%20%20&lib=combine&source=TRANSFAC) | 847 | - | gcAACCGacctcg | Petunia | TRANSFAC |
| [MYB.Ph3](http://plantpan.mbc.nctu.edu.tw/tf_id_search.php?tfid=%20P$MYBPH3_02%20%20%20%20%20%20%20%20%20%20%20&lib=combine&source=TRANSFAC) | 225 | - | agAACTAtgggcc | Petunia | TRANSFAC |
| [LIM1](http://plantpan.mbc.nctu.edu.tw/tf_id_search.php?tfid=%20P$LIM1_01%20%20%20%20%20%20%20%20%20%20%20%20%20&lib=combine&source=TRANSFAC) | 671 | + | CCACCgaaatgc | Tobacco | TRANSFAC |
| [TEIL](http://plantpan.mbc.nctu.edu.tw/tf_id_search.php?tfid=%20P$TEIL_01%20%20%20%20%20%20%20%20%20%20%20%20%20&lib=combine&source=TRANSFAC) | 211 | - | aaaTACAT | Tobacco | TRANSFAC |
| [TEIL](http://plantpan.mbc.nctu.edu.tw/tf_id_search.php?tfid=%20P$TEIL_01%20%20%20%20%20%20%20%20%20%20%20%20%20&lib=combine&source=TRANSFAC) | 1153 | + | ATGTAtta | Tobacco | TRANSFAC |
| [ABI4](http://plantpan.mbc.nctu.edu.tw/tf_id_search.php?tfid=P$ABI4&lib=combine&source=JASPER) | 966 | + | CCGCTTCT | Maize | JASPER |
| [ABRELATERD1](http://plantpan.mbc.nctu.edu.tw/tf_id_search.php?tfid=P$ABRELATERD1&lib=combine&source=PLACE) | 706 | + | ACGTG | Arabidopsis | PLACE |
| [ABRERATCAL](http://plantpan.mbc.nctu.edu.tw/tf_id_search.php?tfid=P$ABRERATCAL&lib=combine&source=PLACE) | 705 | + | AACGTGT | Arabidopsis | PLACE |
| [ABRERATCAL](http://plantpan.mbc.nctu.edu.tw/tf_id_search.php?tfid=P$ABRERATCAL&lib=combine&source=PLACE) | 974 | + | AACGCGT | Arabidopsis | PLACE |
| [ABRERATCAL](http://plantpan.mbc.nctu.edu.tw/tf_id_search.php?tfid=P$ABRERATCAL&lib=combine&source=PLACE) | 975 | - | ACGCGTG | Arabidopsis | PLACE |
| [ACGTATERD1](http://plantpan.mbc.nctu.edu.tw/tf_id_search.php?tfid=P$ACGTATERD1&lib=combine&source=PLACE) | 1225 | + | ACGT | Arabidopsis | PLACE |
| [ACGTATERD1](http://plantpan.mbc.nctu.edu.tw/tf_id_search.php?tfid=P$ACGTATERD1&lib=combine&source=PLACE) | 1225 | - | ACGT | Arabidopsis | PLACE |
| [ACGTATERD1](http://plantpan.mbc.nctu.edu.tw/tf_id_search.php?tfid=P$ACGTATERD1&lib=combine&source=PLACE) | 1437 | +/- | ACGT | Arabidopsis | PLACE |
| [ACGTATERD1](http://plantpan.mbc.nctu.edu.tw/tf_id_search.php?tfid=P$ACGTATERD1&lib=combine&source=PLACE) | 151 | +/- | ACGT | Arabidopsis | PLACE |
| [ACGTATERD1](http://plantpan.mbc.nctu.edu.tw/tf_id_search.php?tfid=P$ACGTATERD1&lib=combine&source=PLACE) | 706 | +/- | ACGT | Arabidopsis | PLACE |
| [ACGTATERD1](http://plantpan.mbc.nctu.edu.tw/tf_id_search.php?tfid=P$ACGTATERD1&lib=combine&source=PLACE) | 888 | +/- | ACGT | Arabidopsis | PLACE |
| [AGCBOXNPGLB](http://plantpan.mbc.nctu.edu.tw/tf_id_search.php?tfid=P$AGCBOXNPGLB&lib=combine&source=PLACE) | 844 | +/- | AGCCGCC | tobacco/Arabidopsis | PLACE |
| [AP1](http://plantpan.mbc.nctu.edu.tw/tf_id_search.php?tfid=P$AP1&lib=combine&source=AGRIS) | 1095 | + | TTTTTGG | Arabidopsis | AGRIS |
| [AP1](http://plantpan.mbc.nctu.edu.tw/tf_id_search.php?tfid=P$AP1&lib=combine&source=AGRIS) | 1261 | + | TTTTTAG | Arabidopsis | AGRIS |
| [AP1](http://plantpan.mbc.nctu.edu.tw/tf_id_search.php?tfid=P$AP1&lib=combine&source=AGRIS) | 422 | + | TTTTTGG | Arabidopsis | AGRIS |
| [AP1](http://plantpan.mbc.nctu.edu.tw/tf_id_search.php?tfid=P$AP1&lib=combine&source=AGRIS) | 880 | - | CCAAAAA | Arabidopsis | AGRIS |
| [ARR10](http://plantpan.mbc.nctu.edu.tw/tf_id_search.php?tfid=P$ARR10&lib=combine&source=JASPER) | 1194 | - | AAAAATCT | Arabidopsis | JASPER |
| [ARR10](http://plantpan.mbc.nctu.edu.tw/tf_id_search.php?tfid=P$ARR10&lib=combine&source=JASPER) | 355 | + | AGATTATA | Arabidopsis | JASPER |
| [ARR10](http://plantpan.mbc.nctu.edu.tw/tf_id_search.php?tfid=P$ARR10&lib=combine&source=JASPER) | 757 | + | AGATTTGA | Arabidopsis | JASPER |
| [ARR10](http://plantpan.mbc.nctu.edu.tw/tf_id_search.php?tfid=P$ARR10&lib=combine&source=JASPER) | 822 | - | TCAAATCT | Arabidopsis | JASPER |
| [Agamous](http://plantpan.mbc.nctu.edu.tw/tf_id_search.php?tfid=P$Agamous&lib=combine&source=JASPER) | 397 | - | ACCCCTAATGG | Arabidopsis | JASPER |
| [BIHD1OS](http://plantpan.mbc.nctu.edu.tw/tf_id_search.php?tfid=P$BIHD1OS&lib=combine&source=PLACE) | 1271 | - | TGACA | rice | PLACE |
| [BIHD1OS](http://plantpan.mbc.nctu.edu.tw/tf_id_search.php?tfid=P$BIHD1OS&lib=combine&source=PLACE) | 140 | - | TGACA | rice | PLACE |
| [BIHD1OS](http://plantpan.mbc.nctu.edu.tw/tf_id_search.php?tfid=P$BIHD1OS&lib=combine&source=PLACE) | 1594 | - | TGACA | rice | PLACE |
| [BIHD1OS](http://plantpan.mbc.nctu.edu.tw/tf_id_search.php?tfid=P$BIHD1OS&lib=combine&source=PLACE) | 321 | - | TGACA | rice | PLACE |
| [BIHD1OS](http://plantpan.mbc.nctu.edu.tw/tf_id_search.php?tfid=P$BIHD1OS&lib=combine&source=PLACE) | 46 | - | TGACA | rice | PLACE |
| [BIHD1OS](http://plantpan.mbc.nctu.edu.tw/tf_id_search.php?tfid=P$BIHD1OS&lib=combine&source=PLACE) | 762 | - | TGACA | rice | PLACE |
| [BIHD1OS](http://plantpan.mbc.nctu.edu.tw/tf_id_search.php?tfid=P$BIHD1OS&lib=combine&source=PLACE) | 793 | - | TGACA | rice | PLACE |
| [BIHD1OS](http://plantpan.mbc.nctu.edu.tw/tf_id_search.php?tfid=P$BIHD1OS&lib=combine&source=PLACE) | 820 | + | TGTCA | rice | PLACE |
| [BOXCPSAS1](http://plantpan.mbc.nctu.edu.tw/tf_id_search.php?tfid=P$BOXCPSAS1&lib=combine&source=PLACE) | 1526 | + | CTCCCAC | pea | PLACE |
| [Bellringer](http://plantpan.mbc.nctu.edu.tw/tf_id_search.php?tfid=P$Bellringer&lib=combine&source=AGRIS) | 554 | - | TTTAATTT | Arabidopsis | AGRIS |
| [C1MOTIFZMBZ2](http://plantpan.mbc.nctu.edu.tw/tf_id_search.php?tfid=P$C1MOTIFZMBZ2&lib=combine&source=PLACE) | 1000 | + | CGAACCA | maize | PLACE |
| [C1MOTIFZMBZ2](http://plantpan.mbc.nctu.edu.tw/tf_id_search.php?tfid=P$C1MOTIFZMBZ2&lib=combine&source=PLACE) | 1242 | + | TTAACCA | maize | PLACE |
| [C8GCARGAT](http://plantpan.mbc.nctu.edu.tw/tf_id_search.php?tfid=P$C8GCARGAT&lib=combine&source=PLACE) | 1091 | +/- | CATTTTTTTG | Arabidopsis | PLACE |
| [C8GCARGAT](http://plantpan.mbc.nctu.edu.tw/tf_id_search.php?tfid=P$C8GCARGAT&lib=combine&source=PLACE) | 1134 | +/- | CTAAAATTTG | Arabidopsis | PLACE |
| [C8GCARGAT](http://plantpan.mbc.nctu.edu.tw/tf_id_search.php?tfid=P$C8GCARGAT&lib=combine&source=PLACE) | 918 | +/- | CATTTTATAG | Arabidopsis | PLACE |
| [CAATBOX1](http://plantpan.mbc.nctu.edu.tw/tf_id_search.php?tfid=P$CAATBOX1&lib=combine&source=PLACE) | 100 | + | CAAT | pea | PLACE |
| [CAATBOX1](http://plantpan.mbc.nctu.edu.tw/tf_id_search.php?tfid=P$CAATBOX1&lib=combine&source=PLACE) | 1043 | - | ATTG | pea | PLACE |
| [CAATBOX1](http://plantpan.mbc.nctu.edu.tw/tf_id_search.php?tfid=P$CAATBOX1&lib=combine&source=PLACE) | 1456 | + | CAAT | pea | PLACE |
| [CAATBOX1](http://plantpan.mbc.nctu.edu.tw/tf_id_search.php?tfid=P$CAATBOX1&lib=combine&source=PLACE) | 273 | + | CAAT | pea | PLACE |
| [CAATBOX1](http://plantpan.mbc.nctu.edu.tw/tf_id_search.php?tfid=P$CAATBOX1&lib=combine&source=PLACE) | 34 | + | CAAT | pea | PLACE |
| [CAATBOX1](http://plantpan.mbc.nctu.edu.tw/tf_id_search.php?tfid=P$CAATBOX1&lib=combine&source=PLACE) | 380 | - | ATTG | pea | PLACE |
| [CAATBOX1](http://plantpan.mbc.nctu.edu.tw/tf_id_search.php?tfid=P$CAATBOX1&lib=combine&source=PLACE) | 419 | + | CAAT | pea | PLACE |
| [CAATBOX1](http://plantpan.mbc.nctu.edu.tw/tf_id_search.php?tfid=P$CAATBOX1&lib=combine&source=PLACE) | 434 | + | CAAT | pea | PLACE |
| [CAATBOX1](http://plantpan.mbc.nctu.edu.tw/tf_id_search.php?tfid=P$CAATBOX1&lib=combine&source=PLACE) | 457 | - | ATTG | pea | PLACE |
| [CAATBOX1](http://plantpan.mbc.nctu.edu.tw/tf_id_search.php?tfid=P$CAATBOX1&lib=combine&source=PLACE) | 481 | - | ATTG | pea | PLACE |
| [CAATBOX1](http://plantpan.mbc.nctu.edu.tw/tf_id_search.php?tfid=P$CAATBOX1&lib=combine&source=PLACE) | 512 | - | ATTG | pea | PLACE |
| [CAATBOX1](http://plantpan.mbc.nctu.edu.tw/tf_id_search.php?tfid=P$CAATBOX1&lib=combine&source=PLACE) | 614 | - | ATTG | pea | PLACE |
| [CAATBOX1](http://plantpan.mbc.nctu.edu.tw/tf_id_search.php?tfid=P$CAATBOX1&lib=combine&source=PLACE) | 688 | - | ATTG | pea | PLACE |
| [CAATBOX1](http://plantpan.mbc.nctu.edu.tw/tf_id_search.php?tfid=P$CAATBOX1&lib=combine&source=PLACE) | 941 | + | CAAT | pea | PLACE |
| [CARGCW8GAT](http://plantpan.mbc.nctu.edu.tw/tf_id_search.php?tfid=P$CARGCW8GAT&lib=combine&source=PLACE) | 1091 | +/- | CATTTTTTTG | Arabidopsis | PLACE |
| [CARGCW8GAT](http://plantpan.mbc.nctu.edu.tw/tf_id_search.php?tfid=P$CARGCW8GAT&lib=combine&source=PLACE) | 1134 | +/- | CTAAAATTTG | Arabidopsis | PLACE |
| [CARGCW8GAT](http://plantpan.mbc.nctu.edu.tw/tf_id_search.php?tfid=P$CARGCW8GAT&lib=combine&source=PLACE) | 918 | +/- | CATTTTATAG | Arabidopsis | PLACE |
| [CBFHV](http://plantpan.mbc.nctu.edu.tw/tf_id_search.php?tfid=P$CBFHV&lib=combine&source=PLACE) | 1417 | - | GTCGAT | barley | PLACE |
| [CBFHV](http://plantpan.mbc.nctu.edu.tw/tf_id_search.php?tfid=P$CBFHV&lib=combine&source=PLACE) | 855 | + | ACCGAC | barley | PLACE |
| [CBFHV](http://plantpan.mbc.nctu.edu.tw/tf_id_search.php?tfid=P$CBFHV&lib=combine&source=PLACE) | 875 | + | GCCGAC | barley | PLACE |
| [CCA1](http://plantpan.mbc.nctu.edu.tw/tf_id_search.php?tfid=P$CCA1&lib=combine&source=AGRIS) | 1194 | + | AAAAATCT | Arabidopsis | AGRIS |
| [CGCGBOXAT](http://plantpan.mbc.nctu.edu.tw/tf_id_search.php?tfid=P$CGCGBOXAT&lib=combine&source=PLACE) | 975 | +/- | ACGCGT | Arabidopsis | PLACE |
| [CIACADIANLELHC](http://plantpan.mbc.nctu.edu.tw/tf_id_search.php?tfid=P$CIACADIANLELHC&lib=combine&source=PLACE) | 981 | - | GATTCGCTTG | tomato | PLACE |
| [CMSRE1IBSPOA](http://plantpan.mbc.nctu.edu.tw/tf_id_search.php?tfid=P$CMSRE1IBSPOA&lib=combine&source=potato) | 374 | - | CCGTCCA | sweet | potato |
| [CPBCSPOR](http://plantpan.mbc.nctu.edu.tw/tf_id_search.php?tfid=P$CPBCSPOR&lib=combine&source=PLACE) | 1486 | + | TATTAG | cucumber | PLACE |
| [CPBCSPOR](http://plantpan.mbc.nctu.edu.tw/tf_id_search.php?tfid=P$CPBCSPOR&lib=combine&source=PLACE) | 226 | + | TATTAG | cucumber | PLACE |
| [DPBFCOREDCDC3](http://plantpan.mbc.nctu.edu.tw/tf_id_search.php?tfid=P$DPBFCOREDCDC3&lib=combine&source=PLACE) | 1291 | + | ACACCAG | carrot/Arabidopsis | PLACE |
| [DPBFCOREDCDC3](http://plantpan.mbc.nctu.edu.tw/tf_id_search.php?tfid=P$DPBFCOREDCDC3&lib=combine&source=PLACE) | 728 | - | CAGGTGT | carrot/Arabidopsis | PLACE |
| [DRE2COREZMRAB17](http://plantpan.mbc.nctu.edu.tw/tf_id_search.php?tfid=P$DRE2COREZMRAB17&lib=combine&source=PLACE) | 855 | + | ACCGAC | maize | PLACE |
| [DRECRTCOREAT](http://plantpan.mbc.nctu.edu.tw/tf_id_search.php?tfid=P$DRECRTCOREAT&lib=combine&source=PLACE) | 855 | + | ACCGAC | rice/maize/sunflower | PLACE |
| [DRECRTCOREAT](http://plantpan.mbc.nctu.edu.tw/tf_id_search.php?tfid=P$DRECRTCOREAT&lib=combine&source=PLACE) | 875 | + | GCCGAC | rice/maize/sunflower | PLACE |
| [E2FCONSENSUS](http://plantpan.mbc.nctu.edu.tw/tf_id_search.php?tfid=P$E2FCONSENSUS&lib=combine&source=PLACE) | 1062 | + | TTTGCCGG | Arabidopsis/tobacco/rice | PLACE |
| [ERF1](http://plantpan.mbc.nctu.edu.tw/tf_id_search.php?tfid=P$ERF1&lib=combine&source=AGRIS) | 845 | + | GCCGCC | Arabidopsis | AGRIS |
| [GARE2OSREP1](http://plantpan.mbc.nctu.edu.tw/tf_id_search.php?tfid=P$GARE2OSREP1&lib=combine&source=PLACE) | 1435 | + | TAACGTA | rice | PLACE |
| [GATABOX](http://plantpan.mbc.nctu.edu.tw/tf_id_search.php?tfid=P$GATABOX&lib=combine&source=PLACE) | 1115 | - | TATC | petunia/Arabidopsis/rice | PLACE |
| [GATABOX](http://plantpan.mbc.nctu.edu.tw/tf_id_search.php?tfid=P$GATABOX&lib=combine&source=PLACE) | 1410 | - | TATC | petunia/Arabidopsis/rice | PLACE |
| [GATABOX](http://plantpan.mbc.nctu.edu.tw/tf_id_search.php?tfid=P$GATABOX&lib=combine&source=PLACE) | 1464 | + | GATA | petunia/Arabidopsis/rice | PLACE |
| [GATABOX](http://plantpan.mbc.nctu.edu.tw/tf_id_search.php?tfid=P$GATABOX&lib=combine&source=PLACE) | 251 | - | TATC | petunia/Arabidopsis/rice | PLACE |
| [GATABOX](http://plantpan.mbc.nctu.edu.tw/tf_id_search.php?tfid=P$GATABOX&lib=combine&source=PLACE) | 460 | + | GATA | petunia/Arabidopsis/rice | PLACE |
| [GATABOX](http://plantpan.mbc.nctu.edu.tw/tf_id_search.php?tfid=P$GATABOX&lib=combine&source=PLACE) | 590 | - | TATC | petunia/Arabidopsis/rice | PLACE |
| [GATABOX](http://plantpan.mbc.nctu.edu.tw/tf_id_search.php?tfid=P$GATABOX&lib=combine&source=PLACE) | 633 | - | TATC | petunia/Arabidopsis/rice | PLACE |
| [GATABOX](http://plantpan.mbc.nctu.edu.tw/tf_id_search.php?tfid=P$GATABOX&lib=combine&source=PLACE) | 786 | - | TATC | petunia/Arabidopsis/rice | PLACE |
| [GATABOX](http://plantpan.mbc.nctu.edu.tw/tf_id_search.php?tfid=P$GATABOX&lib=combine&source=PLACE) | 82 | + | GATA | petunia/Arabidopsis/rice | PLACE |
| [GATABOX](http://plantpan.mbc.nctu.edu.tw/tf_id_search.php?tfid=P$GATABOX&lib=combine&source=PLACE) | 91 | + | GATA | petunia/Arabidopsis/rice | PLACE |
| [GBF5](http://plantpan.mbc.nctu.edu.tw/tf_id_search.php?tfid=P$GBF5&lib=combine&source=AGRIS) | 1467 | - | ATGAGT | Arabidopsis | AGRIS |
| [GCCCORE](http://plantpan.mbc.nctu.edu.tw/tf_id_search.php?tfid=P$GCCCORE&lib=combine&source=PLACE) | 845 | + | GCCGCC | Arabidopsis/tomato | PLACE |
| [GT1CONSENSUS](http://plantpan.mbc.nctu.edu.tw/tf_id_search.php?tfid=P$GT1CONSENSUS&lib=combine&source=PLACE) | 1026 | + | GAAAAT | pea/oat/rice/tobacco/Arabidopsis | PLACE |
| [GT1CONSENSUS](http://plantpan.mbc.nctu.edu.tw/tf_id_search.php?tfid=P$GT1CONSENSUS&lib=combine&source=PLACE) | 1052 | - | TTTTTC | pea/oat/rice/tobacco/Arabidopsis | PLACE |
| [GT1CONSENSUS](http://plantpan.mbc.nctu.edu.tw/tf_id_search.php?tfid=P$GT1CONSENSUS&lib=combine&source=PLACE) | 1077 | + | GAAAAA | pea/oat/rice/tobacco/Arabidopsis | PLACE |
| [GT1CONSENSUS](http://plantpan.mbc.nctu.edu.tw/tf_id_search.php?tfid=P$GT1CONSENSUS&lib=combine&source=PLACE) | 1082 | - | ATTTCC | pea/oat/rice/tobacco/Arabidopsis | PLACE |
| [GT1CONSENSUS](http://plantpan.mbc.nctu.edu.tw/tf_id_search.php?tfid=P$GT1CONSENSUS&lib=combine&source=PLACE) | 1113 | - | TTTATC | pea/oat/rice/tobacco/Arabidopsis | PLACE |
| [GT1CONSENSUS](http://plantpan.mbc.nctu.edu.tw/tf_id_search.php?tfid=P$GT1CONSENSUS&lib=combine&source=PLACE) | 1125 | - | TTTTTC | pea/oat/rice/tobacco/Arabidopsis | PLACE |
| [GT1CONSENSUS](http://plantpan.mbc.nctu.edu.tw/tf_id_search.php?tfid=P$GT1CONSENSUS&lib=combine&source=PLACE) | 1249 | + | GAAAAA | pea/oat/rice/tobacco/Arabidopsis | PLACE |
| [GT1CONSENSUS](http://plantpan.mbc.nctu.edu.tw/tf_id_search.php?tfid=P$GT1CONSENSUS&lib=combine&source=PLACE) | 1307 | + | GGTAAT | pea/oat/rice/tobacco/Arabidopsis | PLACE |
| [GT1CONSENSUS](http://plantpan.mbc.nctu.edu.tw/tf_id_search.php?tfid=P$GT1CONSENSUS&lib=combine&source=PLACE) | 1343 | - | ATTTTC | pea/oat/rice/tobacco/Arabidopsis | PLACE |
| [GT1CONSENSUS](http://plantpan.mbc.nctu.edu.tw/tf_id_search.php?tfid=P$GT1CONSENSUS&lib=combine&source=PLACE) | 1344 | - | TTTTCC | pea/oat/rice/tobacco/Arabidopsis | PLACE |
| [GT1CONSENSUS](http://plantpan.mbc.nctu.edu.tw/tf_id_search.php?tfid=P$GT1CONSENSUS&lib=combine&source=PLACE) | 146 | + | GAAAAA | pea/oat/rice/tobacco/Arabidopsis | PLACE |
| [GT1CONSENSUS](http://plantpan.mbc.nctu.edu.tw/tf_id_search.php?tfid=P$GT1CONSENSUS&lib=combine&source=PLACE) | 249 | - | ATTATC | pea/oat/rice/tobacco/Arabidopsis | PLACE |
| [GT1CONSENSUS](http://plantpan.mbc.nctu.edu.tw/tf_id_search.php?tfid=P$GT1CONSENSUS&lib=combine&source=PLACE) | 314 | + | GAAAAA | pea/oat/rice/tobacco/Arabidopsis | PLACE |
| [GT1CONSENSUS](http://plantpan.mbc.nctu.edu.tw/tf_id_search.php?tfid=P$GT1CONSENSUS&lib=combine&source=PLACE) | 338 | - | ATTTTC | pea/oat/rice/tobacco/Arabidopsis | PLACE |
| [GT1CONSENSUS](http://plantpan.mbc.nctu.edu.tw/tf_id_search.php?tfid=P$GT1CONSENSUS&lib=combine&source=PLACE) | 474 | - | TTTTTC | pea/oat/rice/tobacco/Arabidopsis | PLACE |
| [GT1CONSENSUS](http://plantpan.mbc.nctu.edu.tw/tf_id_search.php?tfid=P$GT1CONSENSUS&lib=combine&source=PLACE) | 638 | - | TTTTTC | pea/oat/rice/tobacco/Arabidopsis | PLACE |
| [GT1CONSENSUS](http://plantpan.mbc.nctu.edu.tw/tf_id_search.php?tfid=P$GT1CONSENSUS&lib=combine&source=PLACE) | 639 | - | TTTTCC | pea/oat/rice/tobacco/Arabidopsis | PLACE |
| [GT1CONSENSUS](http://plantpan.mbc.nctu.edu.tw/tf_id_search.php?tfid=P$GT1CONSENSUS&lib=combine&source=PLACE) | 71 | + | GAAAAT | pea/oat/rice/tobacco/Arabidopsis | PLACE |
| [GT1CONSENSUS](http://plantpan.mbc.nctu.edu.tw/tf_id_search.php?tfid=P$GT1CONSENSUS&lib=combine&source=PLACE) | 717 | - | ATTACC | pea/oat/rice/tobacco/Arabidopsis | PLACE |
| [GT1CONSENSUS](http://plantpan.mbc.nctu.edu.tw/tf_id_search.php?tfid=P$GT1CONSENSUS&lib=combine&source=PLACE) | 766 | - | ATTTTC | pea/oat/rice/tobacco/Arabidopsis | PLACE |
| [GT1CONSENSUS](http://plantpan.mbc.nctu.edu.tw/tf_id_search.php?tfid=P$GT1CONSENSUS&lib=combine&source=PLACE) | 797 | - | ATTTTC | pea/oat/rice/tobacco/Arabidopsis | PLACE |
| [GT1CONSENSUS](http://plantpan.mbc.nctu.edu.tw/tf_id_search.php?tfid=P$GT1CONSENSUS&lib=combine&source=PLACE) | 895 | - | TTTTTC | pea/oat/rice/tobacco/Arabidopsis | PLACE |
| [GT1CONSENSUS](http://plantpan.mbc.nctu.edu.tw/tf_id_search.php?tfid=P$GT1CONSENSUS&lib=combine&source=PLACE) | 905 | + | GAAAAT | pea/oat/rice/tobacco/Arabidopsis | PLACE |
| [GT1CONSENSUS](http://plantpan.mbc.nctu.edu.tw/tf_id_search.php?tfid=P$GT1CONSENSUS&lib=combine&source=PLACE) | 91 | + | GATAAA | pea/oat/rice/tobacco/Arabidopsis | PLACE |
| [GT1CONSENSUS](http://plantpan.mbc.nctu.edu.tw/tf_id_search.php?tfid=P$GT1CONSENSUS&lib=combine&source=PLACE) | 934 | - | TTTTTC | pea/oat/rice/tobacco/Arabidopsis | PLACE |
| [GT1CORE](http://plantpan.mbc.nctu.edu.tw/tf_id_search.php?tfid=P$GT1CORE&lib=combine&source=PLACE) | 1035 | + | GGTTAA | pea | PLACE |
| [GT1CORE](http://plantpan.mbc.nctu.edu.tw/tf_id_search.php?tfid=P$GT1CORE&lib=combine&source=PLACE) | 1242 | - | TTAACC | pea | PLACE |
| [GT1CORE](http://plantpan.mbc.nctu.edu.tw/tf_id_search.php?tfid=P$GT1CORE&lib=combine&source=PLACE) | 950 | - | TTAACC | pea | PLACE |
| [GT1MOTIFPSRBCS](http://plantpan.mbc.nctu.edu.tw/tf_id_search.php?tfid=P$GT1MOTIFPSRBCS&lib=combine&source=PLACE) | 1022 | + | GTGTGAAAATAT | pea | PLACE |
| [HMG-1](http://plantpan.mbc.nctu.edu.tw/tf_id_search.php?tfid=P$HMG-1&lib=combine&source=JASPER) | 1447 | + | CTTTTACTC | Pea | JASPER |
| [HMG-1](http://plantpan.mbc.nctu.edu.tw/tf_id_search.php?tfid=P$HMG-1&lib=combine&source=JASPER) | 1500 | + | GGTTTATTC | Pea | JASPER |
| [HMG-1](http://plantpan.mbc.nctu.edu.tw/tf_id_search.php?tfid=P$HMG-1&lib=combine&source=JASPER) | 976 | + | CGCGTGATT | Pea | JASPER |
| [HMG-IY](http://plantpan.mbc.nctu.edu.tw/tf_id_search.php?tfid=P$HMG-IY&lib=combine&source=JASPER) | 1046 | - | GTTTTTTTTTTCATCA | Pea | JASPER |
| [HMG-IY](http://plantpan.mbc.nctu.edu.tw/tf_id_search.php?tfid=P$HMG-IY&lib=combine&source=JASPER) | 1068 | + | GGAGAGGAAGAAAAAT | Pea | JASPER |
| [HMG-IY](http://plantpan.mbc.nctu.edu.tw/tf_id_search.php?tfid=P$HMG-IY&lib=combine&source=JASPER) | 1184 | + | ACAAAAAAAAAAAAAT | Pea | JASPER |
| [HMG-IY](http://plantpan.mbc.nctu.edu.tw/tf_id_search.php?tfid=P$HMG-IY&lib=combine&source=JASPER) | 1583 | - | ACTATTATTCTTGACA | Pea | JASPER |
| [HMG-IY](http://plantpan.mbc.nctu.edu.tw/tf_id_search.php?tfid=P$HMG-IY&lib=combine&source=JASPER) | 202 | + | AAATAGATGTAAAAAA | Pea | JASPER |
| [HMG-IY](http://plantpan.mbc.nctu.edu.tw/tf_id_search.php?tfid=P$HMG-IY&lib=combine&source=JASPER) | 306 | + | AGTGAGAAGAAAAAGT | Pea | JASPER |
| [HMG-IY](http://plantpan.mbc.nctu.edu.tw/tf_id_search.php?tfid=P$HMG-IY&lib=combine&source=JASPER) | 463 | - | ATTTTTTTATTTTTTT | Pea | JASPER |
| [HMG-IY](http://plantpan.mbc.nctu.edu.tw/tf_id_search.php?tfid=P$HMG-IY&lib=combine&source=JASPER) | 465 | - | TTTTTTATTTTTTTCT | Pea | JASPER |
| [HMG-IY](http://plantpan.mbc.nctu.edu.tw/tf_id_search.php?tfid=P$HMG-IY&lib=combine&source=JASPER) | 468 | - | TTTATTTTTTTCTATT | Pea | JASPER |
| [HMG-IY](http://plantpan.mbc.nctu.edu.tw/tf_id_search.php?tfid=P$HMG-IY&lib=combine&source=JASPER) | 500 | - | GCTTCTTTTCTTATTG | Pea | JASPER |
| [HMG-IY](http://plantpan.mbc.nctu.edu.tw/tf_id_search.php?tfid=P$HMG-IY&lib=combine&source=JASPER) | 542 | - | GTTATGTTACTTTTTA | Pea | JASPER |
| [INRNTPSADB](http://plantpan.mbc.nctu.edu.tw/tf_id_search.php?tfid=P$INRNTPSADB&lib=combine&source=PLACE) | 304 | - | AAAGTGAG | tobacco | PLACE |
| [LTRE1HVBLT49](http://plantpan.mbc.nctu.edu.tw/tf_id_search.php?tfid=P$LTRE1HVBLT49&lib=combine&source=PLACE) | 1104 | + | CCGAAA | barley | PLACE |
| [LTRE1HVBLT49](http://plantpan.mbc.nctu.edu.tw/tf_id_search.php?tfid=P$LTRE1HVBLT49&lib=combine&source=PLACE) | 679 | + | CCGAAA | barley | PLACE |
| [LTRE1HVBLT49](http://plantpan.mbc.nctu.edu.tw/tf_id_search.php?tfid=P$LTRE1HVBLT49&lib=combine&source=PLACE) | 768 | - | TTTCGG | barley | PLACE |
| [LTRECOREATCOR15](http://plantpan.mbc.nctu.edu.tw/tf_id_search.php?tfid=P$LTRECOREATCOR15&lib=combine&source=PLACE) | 856 | + | CCGAC | Arabidopsis/rape | PLACE |
| [LTRECOREATCOR15](http://plantpan.mbc.nctu.edu.tw/tf_id_search.php?tfid=P$LTRECOREATCOR15&lib=combine&source=PLACE) | 876 | + | CCGAC | Arabidopsis/rape | PLACE |
| [MYB2CONSENSUSAT](http://plantpan.mbc.nctu.edu.tw/tf_id_search.php?tfid=P$MYB2CONSENSUSAT&lib=combine&source=PLACE) | 1118 | - | CCGTTA | Arabidopsis | PLACE |
| [MYB4](http://plantpan.mbc.nctu.edu.tw/tf_id_search.php?tfid=P$MYB4&lib=combine&source=AGRIS) | 393 | + | AACAACC | Arabidopsis | AGRIS |
| [MYBCOREATCYCB1](http://plantpan.mbc.nctu.edu.tw/tf_id_search.php?tfid=P$MYBCOREATCYCB1&lib=combine&source=PLACE) | 1118 | - | CCGTT | Arabidopsis | PLACE |
| [MYBCOREATCYCB1](http://plantpan.mbc.nctu.edu.tw/tf_id_search.php?tfid=P$MYBCOREATCYCB1&lib=combine&source=PLACE) | 367 | - | CCGTT | Arabidopsis | PLACE |
| [MYBCORE](http://plantpan.mbc.nctu.edu.tw/tf_id_search.php?tfid=P$MYBCORE&lib=combine&source=PLACE) | 1017 | - | TAACAG | Arabidopsis/petunia | PLACE |
| [MYBCORE](http://plantpan.mbc.nctu.edu.tw/tf_id_search.php?tfid=P$MYBCORE&lib=combine&source=PLACE) | 1118 | + | CCGTTA | Arabidopsis/petunia | PLACE |
| [MYBCORE](http://plantpan.mbc.nctu.edu.tw/tf_id_search.php?tfid=P$MYBCORE&lib=combine&source=PLACE) | 853 | - | CAACCG | Arabidopsis/petunia | PLACE |
| [MYBPZM](http://plantpan.mbc.nctu.edu.tw/tf_id_search.php?tfid=P$MYBPZM&lib=combine&source=PLACE) | 1389 | + | CCTACC | maize | PLACE |
| [MYBST1](http://plantpan.mbc.nctu.edu.tw/tf_id_search.php?tfid=P$MYBST1&lib=combine&source=PLACE) | 1115 | - | TATCC | potato | PLACE |
| [MYBST1](http://plantpan.mbc.nctu.edu.tw/tf_id_search.php?tfid=P$MYBST1&lib=combine&source=PLACE) | 1410 | - | TATCC | potato | PLACE |
| [MYCCONSENSUSAT](http://plantpan.mbc.nctu.edu.tw/tf_id_search.php?tfid=P$MYCCONSENSUSAT&lib=combine&source=PLACE) | 1060 | +/- | CATTTG | Arabidopsis | PLACE |
| [MYCCONSENSUSAT](http://plantpan.mbc.nctu.edu.tw/tf_id_search.php?tfid=P$MYCCONSENSUSAT&lib=combine&source=PLACE) | 728 | +/- | CAGGTG | Arabidopsis | PLACE |
| [RYREPEATBNNAPA](http://plantpan.mbc.nctu.edu.tw/tf_id_search.php?tfid=P$RYREPEATBNNAPA&lib=combine&source=PLACE) | 1459 | - | TGCATG | rape | PLACE |
| [SREATMSD](http://plantpan.mbc.nctu.edu.tw/tf_id_search.php?tfid=P$SREATMSD&lib=combine&source=PLACE) | 1114 | + | TTATCC | Arabidopsis | PLACE |
| [TATABOX2](http://plantpan.mbc.nctu.edu.tw/tf_id_search.php?tfid=P$TATABOX2&lib=combine&source=PLACE) | 196 | - | ATTTATA | pea/tobacco/bean | PLACE |
| [TATABOX2](http://plantpan.mbc.nctu.edu.tw/tf_id_search.php?tfid=P$TATABOX2&lib=combine&source=PLACE) | 199 | + | TATAAAT | pea/tobacco/bean | PLACE |
| [TATABOX5](http://plantpan.mbc.nctu.edu.tw/tf_id_search.php?tfid=P$TATABOX5&lib=combine&source=PLACE) | 1014 | - | AAATAA | pea | PLACE |
| [TATABOX5](http://plantpan.mbc.nctu.edu.tw/tf_id_search.php?tfid=P$TATABOX5&lib=combine&source=PLACE) | 1147 | - | AAATAA | pea | PLACE |
| [TATABOX5](http://plantpan.mbc.nctu.edu.tw/tf_id_search.php?tfid=P$TATABOX5&lib=combine&source=PLACE) | 1151 | - | AAATAA | pea | PLACE |
| [TATABOX5](http://plantpan.mbc.nctu.edu.tw/tf_id_search.php?tfid=P$TATABOX5&lib=combine&source=PLACE) | 1163 | + | TTATTT | pea | PLACE |
| [TATABOX5](http://plantpan.mbc.nctu.edu.tw/tf_id_search.php?tfid=P$TATABOX5&lib=combine&source=PLACE) | 1432 | - | AAATAA | pea | PLACE |
| [TATABOX5](http://plantpan.mbc.nctu.edu.tw/tf_id_search.php?tfid=P$TATABOX5&lib=combine&source=PLACE) | 163 | - | AAATAA | pea | PLACE |
| [TATABOX5](http://plantpan.mbc.nctu.edu.tw/tf_id_search.php?tfid=P$TATABOX5&lib=combine&source=PLACE) | 469 | + | TTATTT | pea | PLACE |
| [TATABOX5](http://plantpan.mbc.nctu.edu.tw/tf_id_search.php?tfid=P$TATABOX5&lib=combine&source=PLACE) | 712 | - | AAATAA | pea | PLACE |
| [TATABOX5](http://plantpan.mbc.nctu.edu.tw/tf_id_search.php?tfid=P$TATABOX5&lib=combine&source=PLACE) | 73 | - | AAATAA | pea | PLACE |
| [TBOXATGAPB](http://plantpan.mbc.nctu.edu.tw/tf_id_search.php?tfid=P$TBOXATGAPB&lib=combine&source=PLACE) | 59 | - | CAAAGT | Arabidopsis | PLACE |
| [TELOBOXATEEF1AA1](http://plantpan.mbc.nctu.edu.tw/tf_id_search.php?tfid=P$TELOBOXATEEF1AA1&lib=combine&source=PLACE) | 1496 | - | TTAGGGTTT | Arabidopsis | PLACE |
| [TGBOXATPIN2](http://plantpan.mbc.nctu.edu.tw/tf_id_search.php?tfid=P$TGBOXATPIN2&lib=combine&source=PLACE) | 705 | + | AACGTG | tomato/Arabidopsis | PLACE |
| [UP2ATMSD](http://plantpan.mbc.nctu.edu.tw/tf_id_search.php?tfid=P$UP2ATMSD&lib=combine&source=PLACE) | 1497 | - | TAGGGTTT | Arabidopsis | PLACE |
| [WBBOXPCWRKY1](http://plantpan.mbc.nctu.edu.tw/tf_id_search.php?tfid=P$WBBOXPCWRKY1&lib=combine&source=potato/wheat/barley) | 810 | - | AGTCAAA | sweet | potato/wheat/barley |
| [WBOXNTERF3](http://plantpan.mbc.nctu.edu.tw/tf_id_search.php?tfid=P$WBOXNTERF3&lib=combine&source=PLACE) | 810 | - | AGTCA | tobacco | PLACE |
| [WBOXNTERF3](http://plantpan.mbc.nctu.edu.tw/tf_id_search.php?tfid=P$WBOXNTERF3&lib=combine&source=PLACE) | 838 | + | TGACC | tobacco | PLACE |
| [id1](http://plantpan.mbc.nctu.edu.tw/tf_id_search.php?tfid=P$id1&lib=combine&source=JASPER) | 1185 | - | CAAAAAAAAAAA | Maize | JASPER |
| [id1](http://plantpan.mbc.nctu.edu.tw/tf_id_search.php?tfid=P$id1&lib=combine&source=JASPER) | 473 | + | TTTTTTCTATTG | Maize | JASPER |
|  |  |  |  |  |  |
